# Aqp4a and Trpv4 mediate regulatory cell volume increase for swimming maintenance of marine fish spermatozoa

**DOI:** 10.1007/s00018-024-05341-w

**Published:** 2024-07-06

**Authors:** Júlia Castro-Arnau, François Chauvigné, Trine L. Toft-Bertelsen, Roderick Nigel Finn, Nanna MacAulay, Joan Cerdà

**Affiliations:** 1 Institute of Marine Sciences, Spanish National Research Council (CSIC), Barcelona, 08003 Spain; 2 Institute of Biotechnology and Biomedicine (IBB), Universitat Autònoma de Barcelona, Bellaterra (Barcelona), 08193 Spain; 3 Department of Neuroscience, University of Copenhagen, Copenhagen N, 2200 Denmark; 4 Department of Biological Sciences, University of Bergen, Bergen, 5020 Norway; 5 Present Address: Department of Cell Biology & Physiology, Washington University in St. Louis School of Medicine, St. Louis, MO 63110 USA

**Keywords:** Fish, Sperm, Aquaporin, Regulatory volume increase, Ion channel, Motility

## Abstract

Volume regulation is essential for cell homeostasis and physiological function. Amongst the sensory molecules that have been associated with volume regulation is the transient receptor potential vanilloid 4 (TRPV4), which is a non-selective cation channel that in conjunction with aquaporins, typically controls regulatory volume decrease (RVD). Here we show that the interaction between orthologous AQP4 (Aqp4a) and TRPV4 (Trpv4) is important for regulatory volume increase (RVI) in post-activated marine fish spermatozoa under high osmotic stress. Based upon electrophysiological, volumetric, and in vivo and ex vivo functional experiments using the pharmacological and immunological inhibition of Aqp4a and Trpv4 our model suggests that upon ejaculation and exposure to the hypertonic seawater, spermatozoon shrinkage is initially mediated by water efflux through Aqp1aa in the flagellar tail. The shrinkage results in an increase in intracellular Ca^2+^ concentration, and the activation of sperm motility and a Na^+^/K^+^/2Cl^−^ (NKCC1) cotransporter. The activity of NKCC1 is required for the initiation of cell swelling, which secondarily activates the Aqp4a-Trpv4 complex to facilitate the influx of water via Aqp4a-M43 and Ca^2+^ via Trpv4 and L-type channels for the mediation of RVI. The inhibitory experiments show that blocking of each of these events prevents either shrinkage or RVI. Our data thus reveal that post-activated marine fish spermatozoa are capable of initiating RVI under a high hypertonic stress, which is essential for the maintenance of sperm motility.

**Supplementary Information:**

The online version contains supplementary material available at 10.1007/s00018-024-05341-w.

## Introduction

The ability of ejaculated spermatozoa to adapt to the sudden osmotic changes in the extratesticular environment is crucial for motility maintenance and fertilization success. In mammals, the microenvironmental osmolality of the female reproductive tract is lower (~ 310 mOsmol) than in the male tract (~ 415 mOsmol) [1]. Ejaculated mammalian spermatozoa thus experience a mild hypoosmotic shock that induces sperm cell swelling and a subsequent regulatory volume decrease (RVD) response via an ion channel mediated efflux of osmolytes, all of which is crucial for motility activation [2–4]. In both freshwater and marine fishes, however, the spermatozoa experience much larger osmotic stresses during motility activation, which are induced by a rapid change in osmolality from ~ 300 mOsmol in the seminal fluid to either ~ 50 or ~ 1100 mOsmol in freshwater and seawater, respectively [5]. Since fish sperm motility can last several minutes, it is likely that cell volume regulation occurs in relation to hypo- or hypertonic stress to sustain motility. However, although sperm regulatory volume changes have been recorded after osmotic-induced motility activation in freshwater spermatozoa [6–8], no data are currently available for marine teleosts. In addition, the physiological and cellular mechanisms underlying potential cell volume regulatory responses in fish sperm remain unknown.

Mammalian spermatozoa express several transmembrane water channels (aquaporins), such as AQP3, -7, -8 and -11, which have been suggested to play a role in cell volume regulatory responses of the spermatozoon after ejaculation and motility activation, and in the elimination of reactive oxygen species [1, 4, 9–12]. However, most of the available evidence supporting these functions is based on the use of non-specific aquaporin inhibitors or correlation analyses between aquaporin content and subfertile subjects [10, 12, 13]. In addition, in most cases knockout models do not show detrimental effects on sperm morphology or fertility competence [14]. One exception occurs in sperm produced by AQP3 deficient mouse, which despite being motile when exposed to a hypotonic environment, show an impaired ability to regulate cell volume upon entering the uterus and the oviduct, resulting in increased tail bending [3]. Consequently, AQP3 has been suggested to function as an osmosensor or mechanosensor in spermatozoa to detect initial cell volume changes and trigger regulatory volume responses in association with volume-sensitive ion channels or cytoskeletal components [3, 4].

In marine teleosts, such as gilthead seabream (*Sparus aurata*), multiple aquaporin paralogs (Aqp1aa, -1ab1, -7, -8bb and − 10bb) present in the ejaculated spermatozoon are differentially redistributed upon seawater-induced motility activation [15]. In this model, flagellar Aqp1aa mediates bulk water efflux following the hyperosmotic shock in seawater, potentially facilitating rapid cell shrinkage and the observed increase in the intracellular Ca^2+^ concentration ([Ca^2+^]_i_), both of which are necessary to activate flagellar motility [16, 17]. In contrast, peroxide-permeable Aqp8bb is involved in post-activated mitochondrial detoxification to allow ATP production necessary for sustained flagellar contractions [18, 19]. The Aqp1ab1 water channel, and the aquaglyceroporins Aqp7 and -10bb, are each restricted to the head (Aqp7) or the anterior tail (Aqp1ab1 and -10bb) of the spermatozoon, and their immunological inhibition affects the swimming trajectory of post-activated spermatozoa rather than their motility, which mimics sperm activated in the presence of K^+^- or Ca^2+^-selective channel antagonists [15–17]. Thus, Aqp1ab1, -7 and -10bb may be involved in the control of the spermatozoon swimming performance, and/or in the case of Aqp7 and -10bb in the transport of glycerol from the seminal fluid during sperm maturation in the extratesticular spermatic ducts, while they may not play a significant role in cell volume regulatory mechanisms.

Members of the transient receptor potential (TRP) superfamily of ion channels, such as TRPC (Canonical) or TRPV (Vanilloid), function as classical transmembrane Ca^2+^ channels in the mammalian sperm involved in capacitation, the acrosome reaction, sperm migration, and fertilization [20, 21]. The TRPV4 polymodal sensory protein is a non-selective cation channel with a preference for Ca^2+^ that acts as temperature-sensitive ion channel in human sperm [22]. However, this channel also responds to isosmotic or hypotonic-induced cell swelling contributing to RVD in somatic cells, and thus functions as a sensor of abrupt volume changes irrespective of the origin of the cell swelling [23–26]. In astrocytes, salivary gland cells and retinal Müller cells, it has been suggested that the contribution of TRPV4 to the RVD response might include the cooperation and physical interaction with AQP4 [23, 27, 28]. However, in some cell types, such as glial cells, the TRPV4/AQP4 macromolecular complex is not required for RVD, since TRPV4 can respond to cell volume increase in the absence of AQP4 [24, 29, 30].

In marine teleost spermatozoa, recent studies have shown that Trpv4-like channels are present along the tail [17], and that *aqp4a* mRNAs are accumulated in the ejaculated sperm [31]. These observations could be consistent with a role of these channels in cell volume regulatory mechanisms. Based on these findings, we therefore employed the gilthead seabream as a model organism to investigate whether a regulatory volume increase (RVI) response occurs in marine spermatozoa following the initial cell shrinkage associated with the seawater-induced hyperosmotic shock, and the potential cooperative role of Trpv4 and Aqp4a in this mechanism.

## Results

### Seabream spermatozoa elicit an external ion-dependent RVI response after seawater activation of motility

**Fig. 1:**
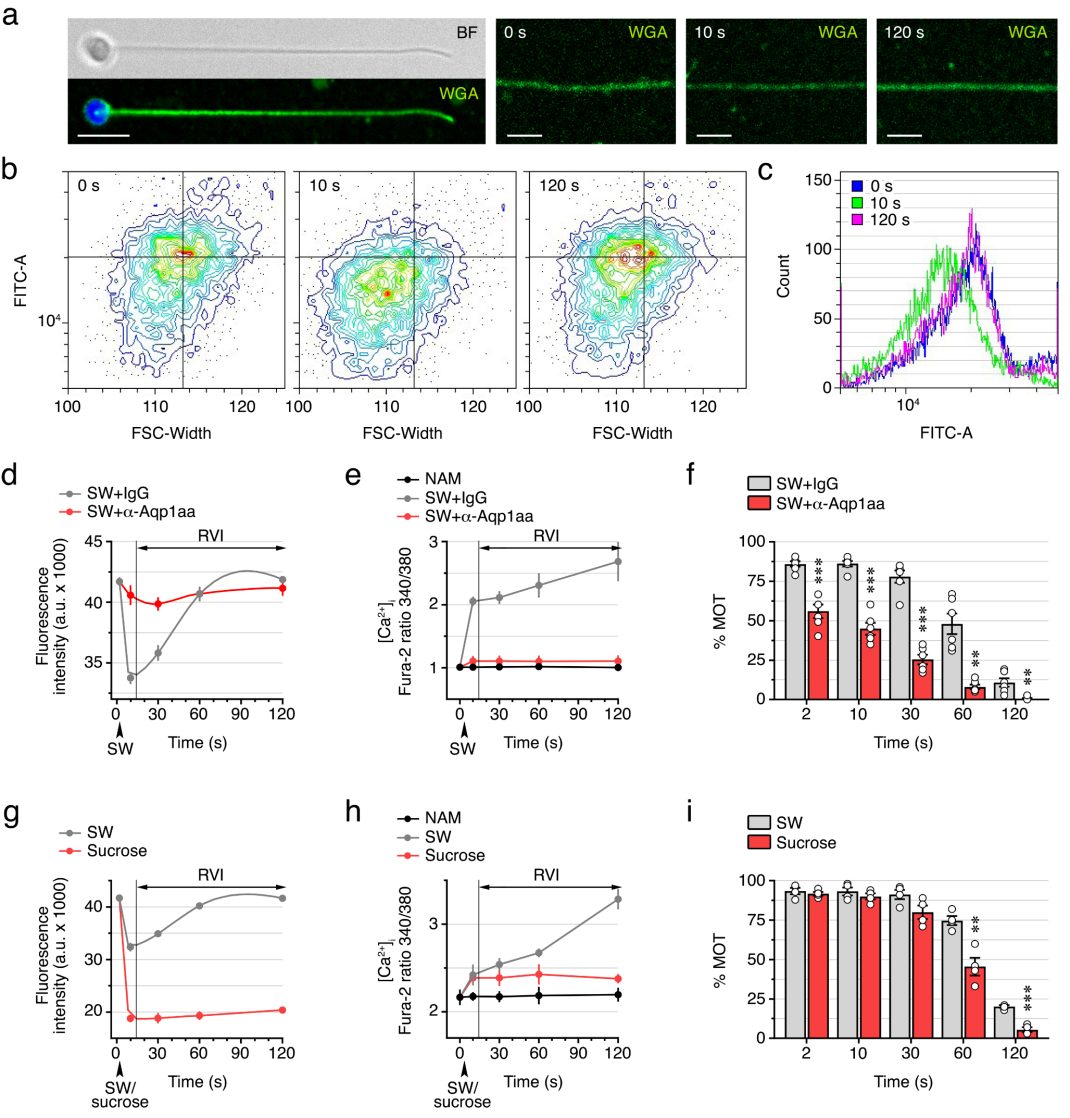
Seabream spermatozoa elicit an RVI response after seawater activation. **a** Bright field (BF) and epifluorescence images of spermatozoa stained with the plasma membrane and nuclear acid fluorescent dyes wheat germ agglutinin (WGA) and DAPI, respectively. Scale bars, 5 μm. The right panels show changes in WGA fluorescence intensity in the flagellum of the spermatozoon before (0 s) and after activation in SW for 10 and 120 s. **b-c** Analysis of spermatozoon volume changes by cell cytometry using WGA before and after activation as in **a**. A shift in size (FSC-Width) and a decrease in fluorescence intensity (FITC-A), both reflecting a drop in cell volume, are observed at 10 s post activation with respect to time 0, which are recovered at 120 s (**b**). The shrinking observed at 10 s is associated with a decrease in FITC-A (**c**). **d, g** Changes in sperm volume after activation in seawater (SW), in the presence of 200 nM of IgY or the specific seabream Aqp1aa antibody (α-Aqp1aa) (**d**), or in 1.1 M sucrose (**g**). **e, h** Changes in the intracellular Ca^2+^ concentration ([Ca^2+^]i) in immotile spermatozoa maintained in non-activated medium (NAM) or during SW or sucrose activation as in **d, g**. In **d, e, g, h**, the duration of the RVI response is indicated with a horizontal double-ended arrow. **f, i** Percentage of motility of sperm treated as in **d, g**. In **d-i**, data are the mean ± SEM (*n* = 3–6 males, white dots), and were statistically analyzed by an unpaired Student *t*-test (**, *p* < 0.01; ***, *p* < 0.001, with respect to IgY or SW treated sperm)

Since changes in spermatozoon volume occurring at activation and post activation in marine teleosts have not been reported, we initially conducted sequential volumetric measurements of activated and post-activated seabream spermatozoa. Cell volume changes were determined by staining the spermatozoon plasma membrane with the fluorophore-coupled lectin wheat germ agglutinin (WGA), which binds to sialic acid and N-acetylglucosamine residues. The signal driven by WGA is directly proportional to cell volume, and thus suitable for estimating cell size [32]. The Alexa Fluor 488-conjugated WGA consistently stained the spermatozoon flagellum at 0, 10 and 120 s post activation (Fig. 1a). However, the fluorescence intensity seemed to decrease at 10 s post activation, and increase again at 120 s, indicating a transient reduction and subsequent recovery of the sperm volume. To more precisely evaluate the changes in the volume of spermatozoa during seawater activation, we measured WGA fluorescence intensity by cell cytometry. This confirmed that the fluorescence decrease of activated sperm at 10 s is restored at 120 s (Fig. 1b and c). These data therefore indicate that despite facing very high hypertonic stress, seabream spermatozoa can regulate their volume with a classical RVI response.

To establish the major effectors involved in volume changes during the activation and prolongation of sperm motility, cell cytometric experiments, together with fluorometric measurements of the [Ca^2+^]_i_ and the kinematic properties of sperm using computer-assisted sperm analysis (CASA), were carried out. In the first trials, sperm was activated in seawater in the absence or presence of a seabream specific Aqp1aa rabbit antibody (α-Aqp1aa), which is able to reduce the channel water conductance [16]. In IgG-treated control sperm, the data showed a strong decrease in cell volume within the first 10 s upon activation, which was followed by a subsequent RVI response up to a level approaching the original cell volume at 60 s post activation and complete volume recovery at 120 s (Fig. 1d). The initial sperm shrinkage was associated with an elevation of the [Ca^2+^]_i_ and the activation of motility, while a further progressive accumulation of Ca^2+^ occurred concomitant with the RVI process until motility faded (Fig. 1e, f). In contrast, the initial hyperosmotic-induced volume reduction of spermatozoa previously treated with the α-Aqp1aa was greatly reduced, which prevented the surge of the [Ca^2+^]_i_ and impaired the initiation of flagellar motility with respect to control spermatozoa (Fig. 1d-f). In addition, in α-Aqp1aa -treated sperm the post activation increase of [Ca^2+^]_i_ was abolished, and a significantly diminished RVI was observed (Fig. 1d, e).

To further investigate the role of external ions in the RVI mechanism, the sperm was activated in seawater or in 1.1 M sucrose, which can also trigger motility of seabream spermatozoa [17]. Under sucrose conditions, the sperm shrinkage was more profound than in seawater, but induced a [Ca^2+^]_i_ rise similar to that observed in control seawater-activated spermatozoa (Fig. 1g, h). However, the post activation RVI response was almost completely abolished in sucrose (Fig. 1g), and the duration of motility was reduced with respect to the seawater-activated sperm (Fig. 1i).

Altogether, these data confirm that the hyperosmotic-induced shrinkage of seawater-activated spermatozoa is mediated by flagellar Aqp1aa, and suggest that the post activation RVI mechanism to maintain motility requires an influx of external Ca^2+^.

### Teleosts harbour a single TRPV4 ortholog with multiple splice variants

**Fig. 2:**
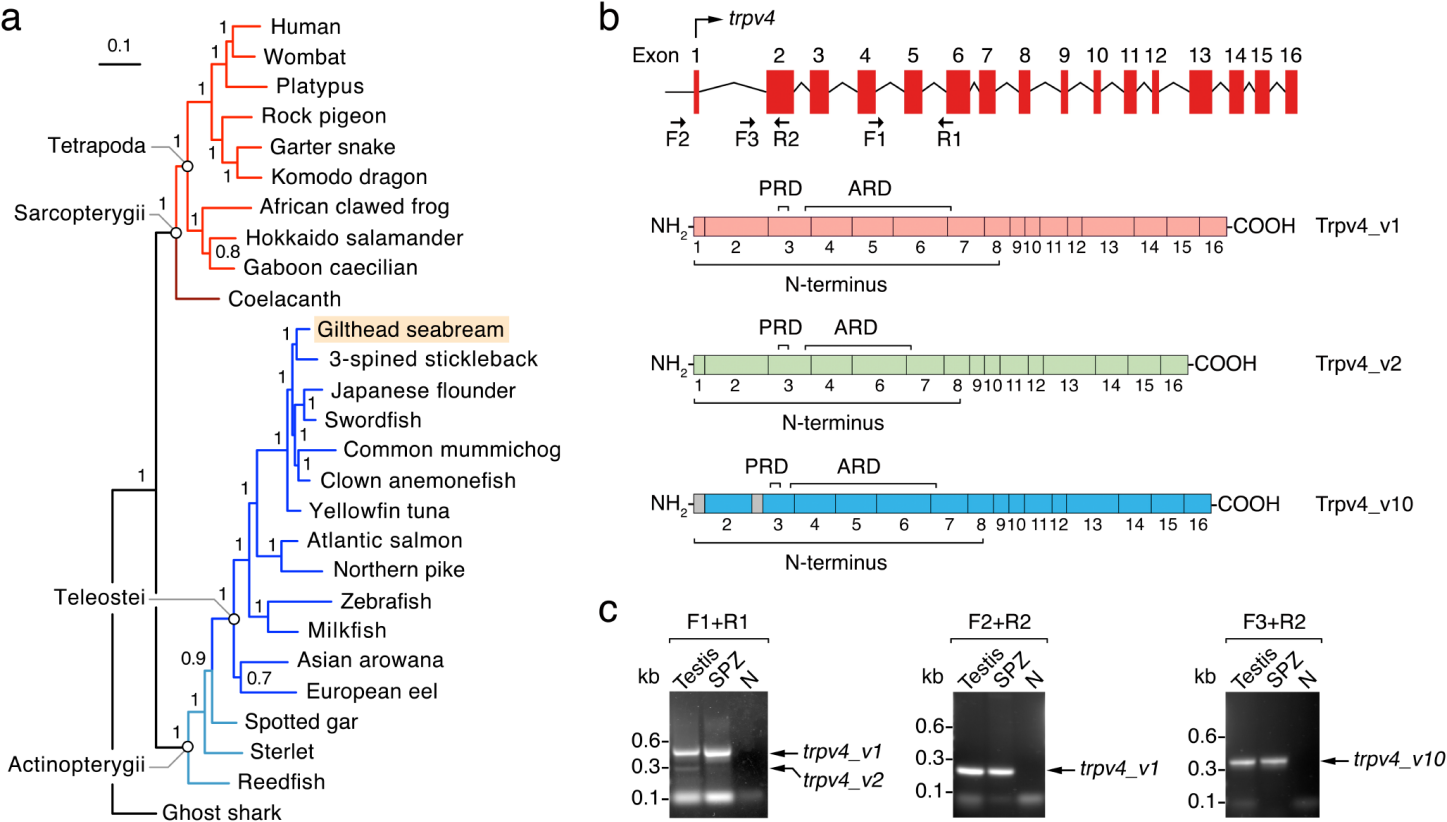
Phylogeny of the TRPV4 in vertebrates and expression of different Trpv4 splice variants in seabream testis and spermatozoa. **a** Bayesian majority rule consensus tree (1 million MCMC generations, aamodel = mixed) of a ClustalX amino acid alignment of Trpv4 protein sequences rooted with the ghost shark Trpv4. The GenBank accession numbers of the sequences are listed in Supplementary Table S1. Bayesian posterior probabilities are shown at each node with the scale bar indicating expected substitution rates per site. **b** Schematic representation of the gilthead seabream *trpv4* exons, and the polypeptides of wild-type Trpv4 (Trpv4_v1) and splice variants Trpv4_v2 and _v10 (Ensembl accession numbers ENSSAUT00010061485.1, ENSSAUT00010061488.1 and ENSSAUT00010061519.1, respectively). The proline-rich domain (PRD) and ankyrin repeat domain (ARD) are shown for each protein. **c** Representative RT-PCR detection of seabream *trpv4_v1, _v2 and _v10* mRNAs in testis and ejaculated spermatozoa (SPZ). The N line is the negative control (absence of RT during cDNA synthesis). The arrows indicate the specific amplified transcripts, and the sizes (kb) of molecular markers are indicated on the left. The positions on the *trpv4* genomic sequence of the different oligonucleotide primers employed are indicated in **b**

We have previously shown that seabream ejaculated spermatozoa express TRPV4-like channels [17]. However, since some teleosts retain a large repertoire of TRPV ion channels due to independent whole genome duplication events and/or ancestral and lineage-specific tandem duplications [33], we first investigated whether duplicated Trpv4 paralogs may exist in seabream and other teleosts. Phylogenetic analyses of TRPV4s confirm that only single orthologs are present in diploid jawed vertebrates (Fig. 2a). The seabream *trpv4* gene is composed of 16 exons, with the encoded protein showing an extensive cytoplasmic N-terminus and several functional domains, including the proline-rich domain (PRD), the ankyrin repeat domain (ARD), the linker domain, the transmembrane domain and the TRP domain (Fig. 2b and Supplementary Fig. S1). However, seabream transcriptome database searching revealed the expression of nine different *trpv4* splice variants in addition to the canonical or wild-type isoform *trpv4_v1* (Ensembl v109: *trpv4-201*, ENSSAUT00010061485, or fSpaAur1.1). Among these isoforms, *trpv4-202* (ENSSAUT00010061488.1), lacking exon 5, and *trpv4-210* (ENSSAUT00010061519.1), showing an alternative translation initiation site and a different exon 1 together with the incorporation of part of intron 2 into exon 3, have the most divergent N-termini with respect to the wild-type, and were termed Trpv4_v2 and Trpv4_v10, respectively (Fig. 2b. Reverse-transcription PCR (RT-PCR) using oligonucleotide primers specific for each isoform showed that the three variants are expressed in the seabream testis, whereas mRNAs encoding Trpv4_v1 and _v10 were the only variants detected in ejaculated spermatozoa (Fig. 2c).

### The Trpv4 and Aqp4a channels are expressed in the seabream sperm tail

**Fig. 3:**
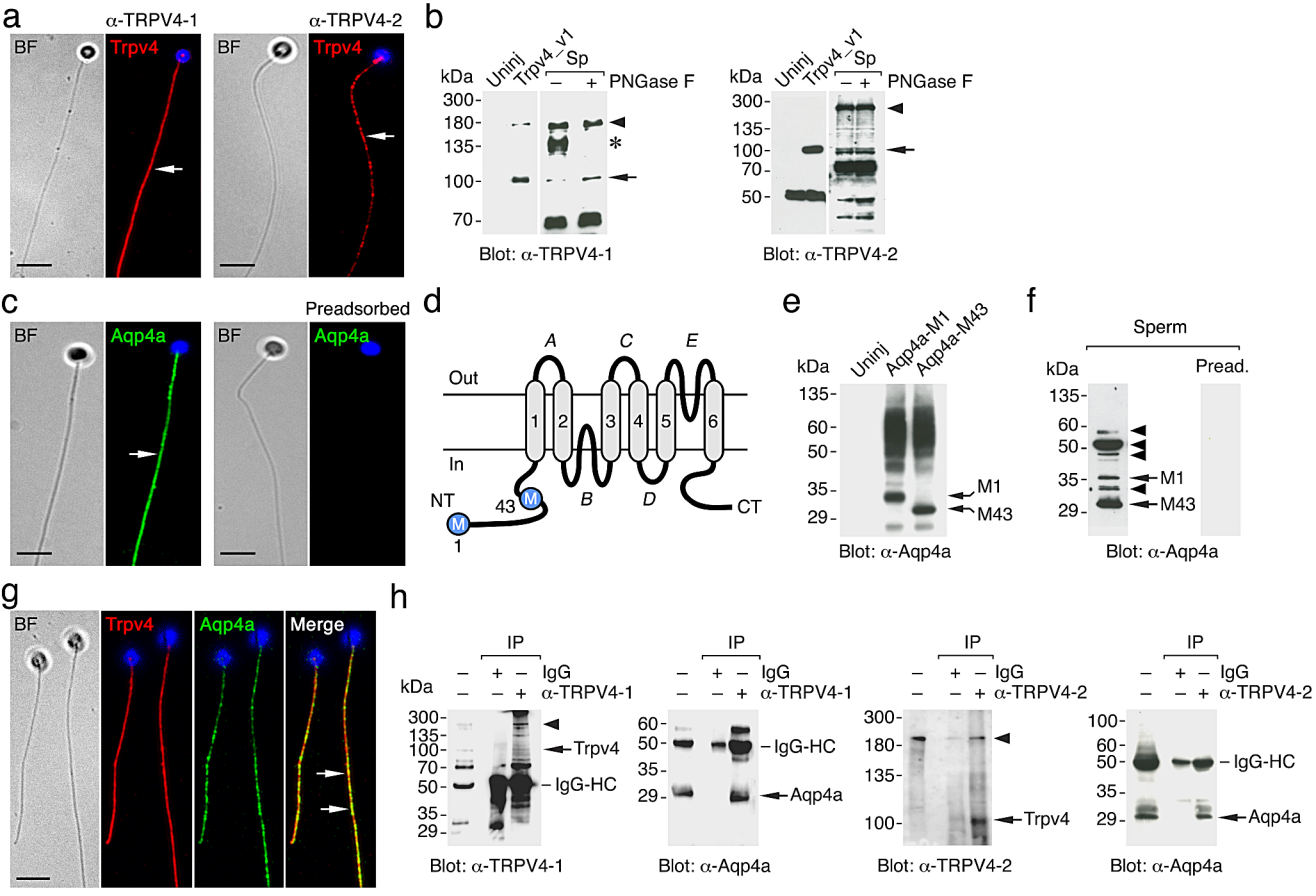
Both Trpv4 and Aqp4a are expressed in seabream spermatozoa. **a** Immunostaining (left panels, bright field [BF]; right panels, epifluorescence images) of Trpv4 in ejaculated spermatozoa using two commercial rabbit antibodies against mammalian TRPV4 (α-TRPV4-1 [Novus Biologicals # NBP2-41262] and α-TRPV4-2 [Invitrogen # OSR00136W]), showing that the Trpv4 immunoreaction (red color) was localized along the flagellum (arrows). The nucleus is counterstained with 4′,6-diamidino-2-phenylindole (DAPI, blue). Scale bars, 5 μm. **b** Immunoblots of total membrane protein extracts from *X. laevis* oocytes uninjected (Uninj.), oocytes injected with cRNA encoding seabream Trpv4_v1, and sperm flagella (Sp), using the two TRPV4 antibodies. Spermatozoa extracts were treated with or without PNGase F (plus or minus) prior to electrophoresis. Arrows indicate monomers, while the asterisks indicate glycosylated forms. The arrowheads indicate other potential post-translational modifications of the ion channel. Molecular mass markers (kDa) are on the left. **c** Immunolocalization of seabream Aqp4a (green) along the tail of the spermatozoa (arrows) using a paralog-specific chicken antibody (α-Aqp4a). The negative controls (right panels) were incubated with the primary antibody preadsorbed with the antigenic peptide. Labels and scale bars as in A. **d** Schematic diagram of the Aqp4a topology depicting the cytoplasmatic N- and C-termini (NT and CT, respectively), the six transmembrane α-helices (1–6), and the five loops (*A-E*). The two translation initiating methionines (M1 and M43) in the NT are shown. **e, f** Immunoblot of *X. laevis* oocytes uninjected (Uninj) or expressing the Aqp4a-M1 or Aqp4a-M43 isoforms (**e**), and of sperm flagella showing the expression of both isoforms (arrows) (**f**). For the latter immunoblot, the α-Aqp4a was preadsorbed with the antigenic peptide before immunoblotting to verify the specificity of the reactive bands. Labels as in **b**. **g** Double immunostaining of Trpv4 and Aqp4a in seabream spermatozoa using the TRPV4-1 and Aqp4a antibodies showing co-localization of both channels (arrows). Labels and scale bars as in **a**. **h** Co-immunoprecipitation of Trpv4 and Aqp4a in ejaculated spermatozoa using either of the two commercial TRPV4 antibodies or immunoglobulin G (IgG) as control. The immunoprecipitates were immunoblotted with the TRPV4 or Aqp4a antibodies as indicated. Labels as in **b**. IgG-HC, IgG heavy chain

To reexamine the expression of Trpv4 in seabream spermatozoa, we used two commercial rabbit polyclonal antibodies against different epitopes of human and mouse TRPV4, which showed 76–79% amino acid sequence identity with the orthologous conserved sequences of seabream Trpv4_v1, _v2 or _v10. Immunofluorescence microscopy with either of the two antibodies showed that Trpv4 was present along the flagellum of spermatozoa (Fig. 3a), in agreement with our earlier study [17]. Immunoblotting of *Xenopus laevis* oocytes expressing the Trpv4_v1, and of the flagellar fraction of spermatozoa, revealed that each of the commercial antisera detected a reactive band with a molecular mass of ~ 100 kDa, consistent with the *in silico* calculated mass of the Trpv4_v1 monomer (99 kDa). This confirmed that both antibodies cross-reacted with the seabream ortholog (Fig. 3b). In some protein extracts, however, additional reactive bands with higher or lower molecular masses were revealed, some of which correspond to peptide-N-glycosidase F-(PNGase F)-sensitive posttranslational modifications of the channel, as reported for human TRPV4 [34], and others possibly being PNGase F-insensitive glycosylated Trpv4, cross-reactive polypeptides or degradation products (Fig. 3b). These data therefore confirmed the expression of Trpv4 in seabream sperm flagella, but because the calculated molecular masses of Trpv4_v1 and _v10 are equivalent, it was not possible to discern whether both isoforms are present in the spermatozoon.

To assess whether Aqp4a is expressed in ejaculated spermatozoa, we employed a previously characterized custom-made chicken antibody raised against the C-terminus of seabream Aqp4a [35]. Immunostaining showed that Aqp4a was also located along the flagellum of spermatozoa as observed for Trpv4, but was not detected when the antiserum was preadsorbed with the immunizing peptide, indicating the specificity of the reaction (Fig. 3c). In mammals, AQP4 exists as two major isoforms, a long isoform (M1) with translation initiation at Met-1, and a shorter isoform (M23) with translation initiation at Met-23 [36]. Similarly, seabream *aqp4a* transcript also contains two distinct translation initiation sites at Met-1 and Met-43, encoding a putative 36-kDa protein isoform with a long N-terminus (Aqp4a-M1), and a 31-kDa short isoform (Aqp4a-M43) (Fig. 3d), which are both recognized by the Aqp4a-specific antibody when expressed in *X. laevis* oocytes (Fig. 3e). Immunoblotting of flagellar proteins detected the two Aqp4a-M1 and -M43 isoforms, although the latter polypeptide band was more intense, indicating that the Aqp4a-M43 variant is predominant in ejaculated spermatozoa (Fig. 3f). The Aqp4a antibody detected additional bands of ~ 32, ~48, ~ 52 and ~ 56 kDa in the flagellar extracts, which could correspond to other Aqp4a variants [37], or posttranslational modifications of the channel, since all reactive bands were no longer observed when using the preadsorbed antiserum (Fig. 3f).

Double immunofluorescence microscopy showed that Trpv4 and Aqp4a co-localized along the spermatozoon flagellum (Fig. 3g), and therefore the possibility of a physical interaction between both channels was further investigated by co-immunoprecipitation. These experiments showed that Aqp4a-M43 could be detected in immunoprecipitated extracts from whole ejaculated spermatozoa using either of the two TRPV4 antibodies (Fig. 3h). This suggests that both channels are physically linked in the spermatozoon flagellum.

### Seabream Trpv4 variants are differentially activated

**Fig. 4:**
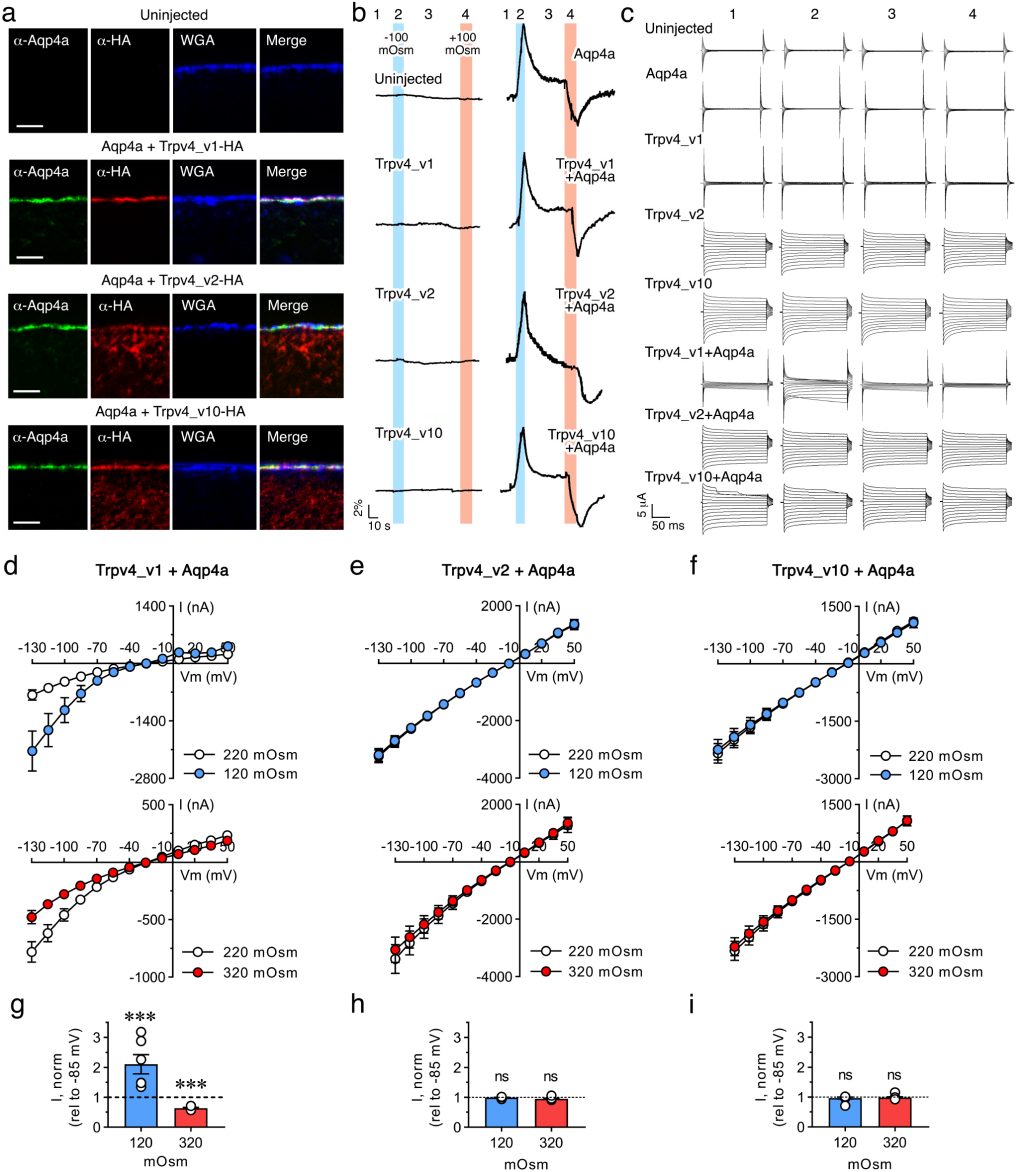
Functional characterization of seabream Trpv4 variants in *X. laevis* oocytes. **a** Representative double immunostaining of uninjected oocytes and oocytes expressing Aqp4a-M43 together with human influenza hemagglutinin (HA)-tagged Trpv4_v1, _v2 or _v10 using a seabream Aqp4a-specific antiserum and anti-HA antibodies. The oocyte plasma membrane was counterstained with wheat germ agglutinin (WGA). Scale bars, 10 μm. **b-c** Representative volume (**b**) and current (**c**) traces obtained from oocytes voltage-clamped at Vm = − 20 mV and challenged with a hypo- or hyperosmotic gradient (− 100 mOsm: blue bars and + 100 mOsm: red bars, respectively). The traces were recorded with a 200-ms step protocol (as indicated by numbers 1–4) from an uninjected oocyte and oocytes expressing either Aqp4a, Trpv4_v1, _v2 or _v10 alone, or Aqp4a together with Trpv4_v1, _v2 or _v10. **d-f** Summarized I/V curves from oocytes expressing Aqp4a plus Tpv4_v1, _v2 or _v10 in control solution (white) or during application of a hyposmotic (blue) or hyperosmotic solution (red). **g-i** Trpv4_v1, _v2 or _v10-mediated current activity at -85 mV obtained after exposure to -100 mOsm (blue) or + 100 mOsm (red) normalized to that obtained in control conditions. In **d-i** panels, values (*n* = 5–9 oocytes; white dots in **g-i**) are presented as mean ± SEM. The paired normalized data in **g-i** were statistically analyzed by one sample *t*-test (***, *p* < 0.001, with respect to the same oocytes at 220 mOsm prior to osmotic challenge; ns, not statistically significant). The controls (uninjected oocytes and oocytes expressing Aqp4a or Tpv4_v1, Trpv4_v2 or Trpv4_v10 alone) and exposed to an isosmotic solution or during application of a hyposmotic or hyperosmotic solution are shown in Supplementary Fig. S2

Activation of rat TRPV4 by cell swelling is dependent on the N-terminus of the ion channel [38]. To investigate whether seabream Trpv4_v1 is also activated by volume changes, as well as potential differential responses of Trpv4 splice variants with different N-termini (Supplementary Fig. S1), the Trpv4_v1, _v2 or _v10 isoforms were heterogeneously expressed in *X. laevis* oocytes together with Aqp4a-M43, and subsequently exposed to osmotic challenges. Immunostaining of human influenza hemagglutinin (HA)-tagged Trpv4 variants in oocytes co-expressing Aqp4a revealed that all the channels were targeted to the plasma membrane, whereas no staining was detected in the uninjected control oocytes (Fig. 4a). However, while the wild-type Trpv4_v1 was only detected in the plasma membrane, the Trpv4_v2 and _v10 variants were also partially retained in the cytoplasm. These experiments demonstrate a close physical relationship of the Trpv4 and Aqp4a membrane proteins and confirmed their successful targeting to the plasma membrane.

To characterize the Trpv4 response to acute volume changes, oocytes were sequentially challenged with osmotic gradients of -100 mOsm (hyposmotic) and + 100 mOsm (hyperosmotic), achieved by removal or addition of 100 mM mannitol from the control solution to respectively decrease or increase the osmolarity, and their volumes sequentially monitored by image analysis. In these experiments, each oocyte was set as its own control, and the effects of the osmotic challenges are shown relative to the basal control currents [38] (Fig. 4b-f). The resultant cell volume response by Trpv4_v1-, _v2- or _v10-expressing oocytes was indistinguishable from that of the uninjected oocytes, whereas oocytes expressing Aqp4a responded to hypo- and hyperosmotic changes, regardless of whether or not the Trpv4 variants were co-expressed (Fig. 4b). This indicated that the cell volume changes responding to the altered osmotic gradient are mediated by Aqp4a.

To determine whether the different Trpv4 variants sensed Aqp4a-mediated cell volume changes, Trpv4-induced current activity during the osmotic challenge was continuously monitored with a conventional two-electrode voltage clamp (Fig. 4c). Oocytes expressing only Aqp4a failed to conduct currents greater than those of uninjected oocytes both in control solution and upon application of an osmotic gradient (Supplementary Fig. S2). However, when the wild-type Trpv4_v1 was co-expressed with Aqp4a, the hyposmotic gradient resulted in swelling and an increased Trpv4_v1-mediated current activity, whereas exposure to the hyperosmotic gradient led to a cell shrinkage-induced current reduction with respect to the basal activity (Fig. 4c, d and g). In contrast, oocytes expressing Trpv4_v2 or _v10 in the absence of Aqp4a showed higher constitutive membrane currents with respect to the controls than Trpv4_v1 (Fig. 4c and Supplementary Fig. S2). In addition, oocytes expressing Aqp4a and Trpv4_v2 or _v10 did not respond with increased current activity upon cell swelling or shrinkage (Fig. 4c, e and f). These data indicate that seabream Trpv4_v1 is sensitive to cell volume changes, and that N-terminal variations of the channel may be involved in dictating its constitutive activity or sensitivity to volume changes.

### Antagonist- and antibody-mediated inhibition of Trpv4 and Aqp4a

**Fig. 5:**
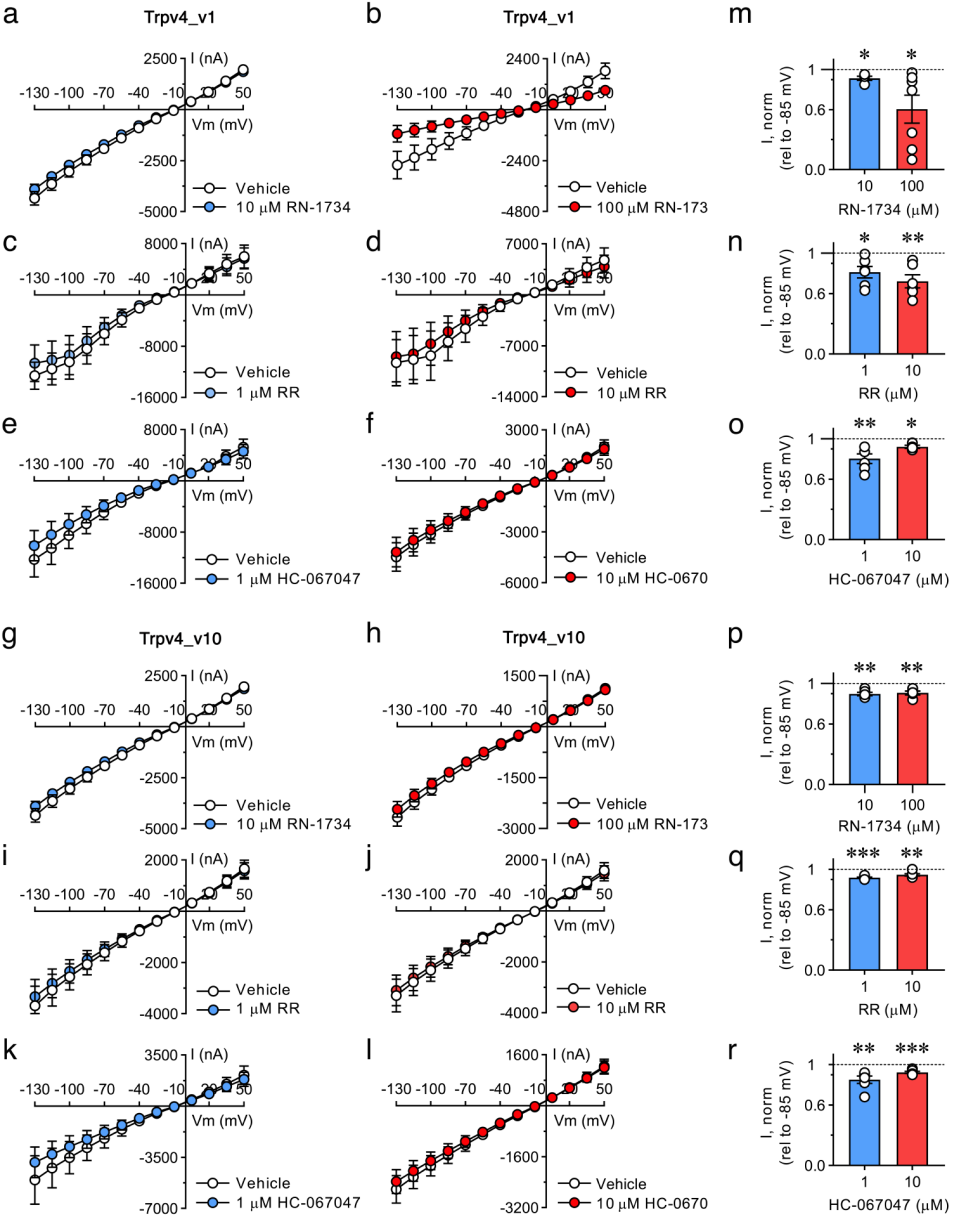
Inhibition of Trpv4_v1 and _v10 with different TRPV4 blockers in *X. laevis oocytes.***a-l** Summarized I/V curves from oocytes expressing either Tpv4_v1 (**a-f**) or _v10 (**g-l**) alone in control solution (white dots) or after 5 min exposure to different TRPV4 blockers at two concentrations, RN-1734 (10 and 100 µM, blue and red dots, respectively), ruthenium red (RR, 1 and 10 µM, blue and red dots, respectively) and HC-067047 (1 and 10 µM, blue and red dots, respectively). **m-r** The Trpv4_v1- (**m-o**) or _v10- (**p-r**) mediated current activity at -85 mV obtained after exposure to the different TRPV4 blockers was normalized to that obtained in control conditions. In all panels, data are presented as mean ± SEM (*n* = 5–6 oocytes; white dots in **m-r**). The paired normalized values were statistically analyzed by one sample *t*-test (*, *p* < 0.05; **, *p* < 0.01; ***, *p* < 0.001, with respect to the same oocytes before treatment with the inhibitors; ns, not statistically significant)

To investigate the physiological function of Trpv4 and Aqp4a during RVI in spermatozoa, we first evaluated the efficiency of different pharmacological and immunological approaches to block the activity of the channels. For Trpv4, we tested three different, well-established TRPV4 antagonists for their effects on the activity of the ion channel. Accordingly, the Trpv4_v1 and _v10, which are the isoforms detected in spermatozoa, were expressed in *X. laevis* oocytes and constitutive current activities were measured after 5 min exposure to the TRPV4 selective antagonists RN-1734 [39] and HC-067047 [40], and the non-selective TRP channel blocker ruthenium red (RR) [41]. Application of RN-1734 resulted in a reduction of the Trpv4_v1 current activity by ~ 9% and ~ 40% with a dose of 10 and 100 µM of the antagonist, respectively (Fig. 5a, b and m). The RR inhibited the Trpv4_v1 by ~ 18% and ~ 28% with 1 and 10 µM, respectively (Fig. 5c, d and n), whereas the HC-067047 showed the same efficiency at blocking the channel (~ 19% and ~ 8% with 1 and 10 µM, respectively) (Fig. 5e, f and o). However, the RN-1734 and RR antagonists were much less effective at reducing the activity of the Trpv4_v10 isoform, since the highest doses of these drugs (100 and 10 µM, respectively) resulted, respectively, in ~ 9% and ~ 5% inhibition (Fig. 5g-j, p and q), while the HC-067047 showed a similar inhibitory potency for Trpv4_v10 and Trpv4_v1 (~ 8% inhibition with 10 µM) (Fig. 5k, l and r). To examine if the treatment time influenced the effect of the TRPV4 antagonists, we incubated oocytes expressing Trpv4_v1 or _v10 with the highest dose of each compound for 1 h (Supplementary Fig. S3). Under these conditions, the RN-1734 and RR only incremented the inhibition of the Trpv4_v10 constitutive current activity up to ~ 33%, while the HC-067047 was able to increase the blockage of the Trpv4_v1 and _v10 channels by ~ 24% and ~ 32%, respectively. Based on these data, the RN-1734 inhibitor appeared to be the most suitable antagonist for blocking the current activity of seabream Trpv4_v1.

**Fig. 6:**
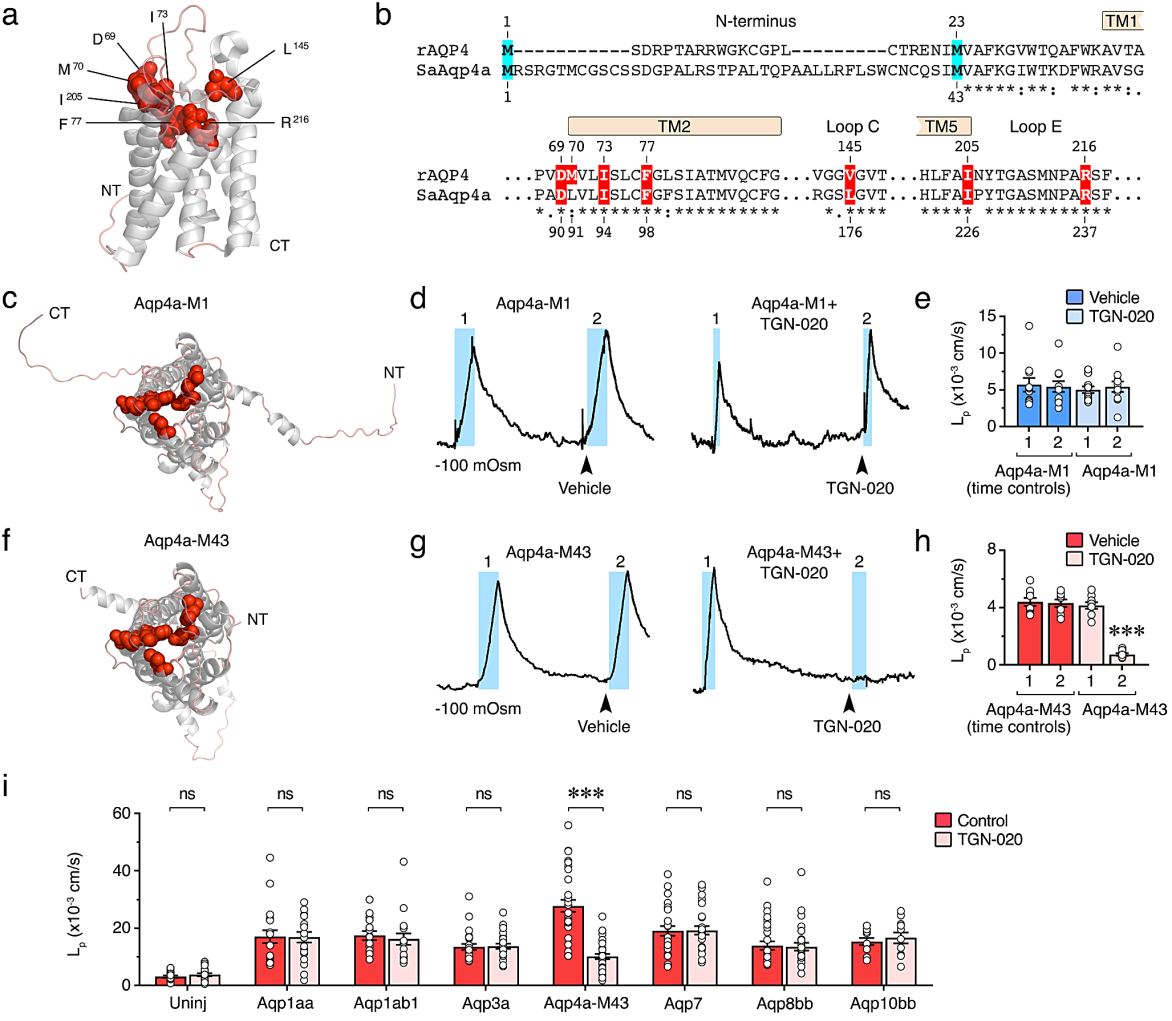
The TGN-020 inhibitor blocks seabream Aqp4a-mediated water permeability in an isoform-specific manner. **a** Three-dimensional structure of crystallized human AQP4 (PDB: 3gd8) cartoon rendered with PyMOL. The molecule is arranged with the extracellular oriented vestibule at the top. The likely binding sites of TGN-020 in rat AQP4 are shown as spacefill (red). NT, amino-terminus; CT, carboxyl terminus. **b** N-terminal and subdomain amino acid alignment of rat AQP4 (rAQP4) and seabream Aqp4a (SaAqp4a) showing that the residues involved in TGN-020 interactions are highly conserved in the fish ortholog. **c, f** Cartoon renders of seabream Aqp4a-M1 (template: A0A4U5UWT1) and Aqp4a-M43 (template: 2zz9) homology modelled via the automatic SWISS-MODEL pipeline. Extracellular views are shown with TGN-020 interacting residues rendered as spacefill (red). Labels as in **a**. **d, g** Representative volume traces of *X. laevis* oocytes expressing seabream Aqp4-M1 or Aqp4a-M43 without (left) or with (right) pretreatment with 20 µM TGN-020 for 5 min prior to recordings. Background blue bars (1 and 2) indicate where a hyposmotic challenge (− 100 mOsm) was abruptly introduced. **e, h** Osmotic water permeability (L_p_) of oocytes treated as in **d** or **g** blue bars (1 and 2). **i** L_p_ of uninjected oocytes or oocytes expressing different seabream aquaporin paralogs without or with 1 h of TGN-020 treatment (shown in pink). in **e, h** and **i**, data are the mean ± SEM (*n* = 11, 9 and 10–32 oocytes/treatment in **e**, **h** and **i**, respectively; white dots), and were statistically analyzed by a paired (**e** and **h**) or unpaired (**i**) Student *t*-test (***, *p* < 0.001; ns, not statistically significant)

To specifically inhibit Aqp4a, we tested the efficiency and selectivity of the antiepileptic molecule 2-(Nicotinamide)-1,3,4-thiadiazole (TGN-020), which has been shown to bind to mammalian AQP4 and act as an inhibitor of the channel water conductance [29, 42–44]. In silico studies using rat AQP4-M23 (rAQP4-M23) as a model have identified the residues D69, M70, I73, F77, V145, I205 and R216, surrounding the extracellular water pore region, as the likely binding sites of TGN-020 [42] (Fig. 6a). These residues are conserved in both seabream Aqp4a isoforms, except for rAQP4 M70 and V145, which are substituted by L91 and L176 in the fish ortholog (Fig. 6b, c and f). We therefore evaluated the effect of TGN-020 on Aqp4a-M1- and -M43-mediated water permeability in oocytes expressing each of the two isoforms, which are equally targeted to the oocyte plasma membrane (Fig. 4a and [35]). The osmotic water permeability (L_p_) of the oocytes was determined following an acute hyposmotic challenge (-100 mOsm) by sequential recording of the oocyte volume changes, using each oocyte as its own control [29]. Exposure to TGN-020 (20 µM, 5 min) or the drug vehicle did not affect the AQP4-M1-dependent oocyte swelling following introduction of the osmotic challenge (Fig. 6d), whereas that mediated by Aqp4a-M43 was completely abolished (Fig. 6g). Thus, the L_p_ of Aqp4a-M1-expressing oocytes before and after TGN-020 was similar (Fig. 6e), while in oocytes expressing Aqp4a-M43 the L_p_ was reduced by ~ 82% (Fig. 6h).

To determine whether the TGN-020 inhibition was selective towards Aqp4a-M43, uninjected oocytes and oocytes expressing Aqp4a-M43 or other aquaporin paralogs present in seabream sperm, such as Aqp1aa, -1ab1, -7, -8bb or -10bb [15], or Aqp3a (unpublished data), were exposed to 20 µM TGN-020 for 1 h and their water permeability assessed after direct exposure to a hyposmotic medium (20 mOsm). The TGN-020 exclusively reduced the water permeability of oocytes expressing Aqp4a-M43 (Fig. 6i), thus showing that the inhibitor selectively and efficiently blocks the Aqp4a-M43 isoform within the group of aquaporins present in seabream spermatozoa.

**Fig. 7:**
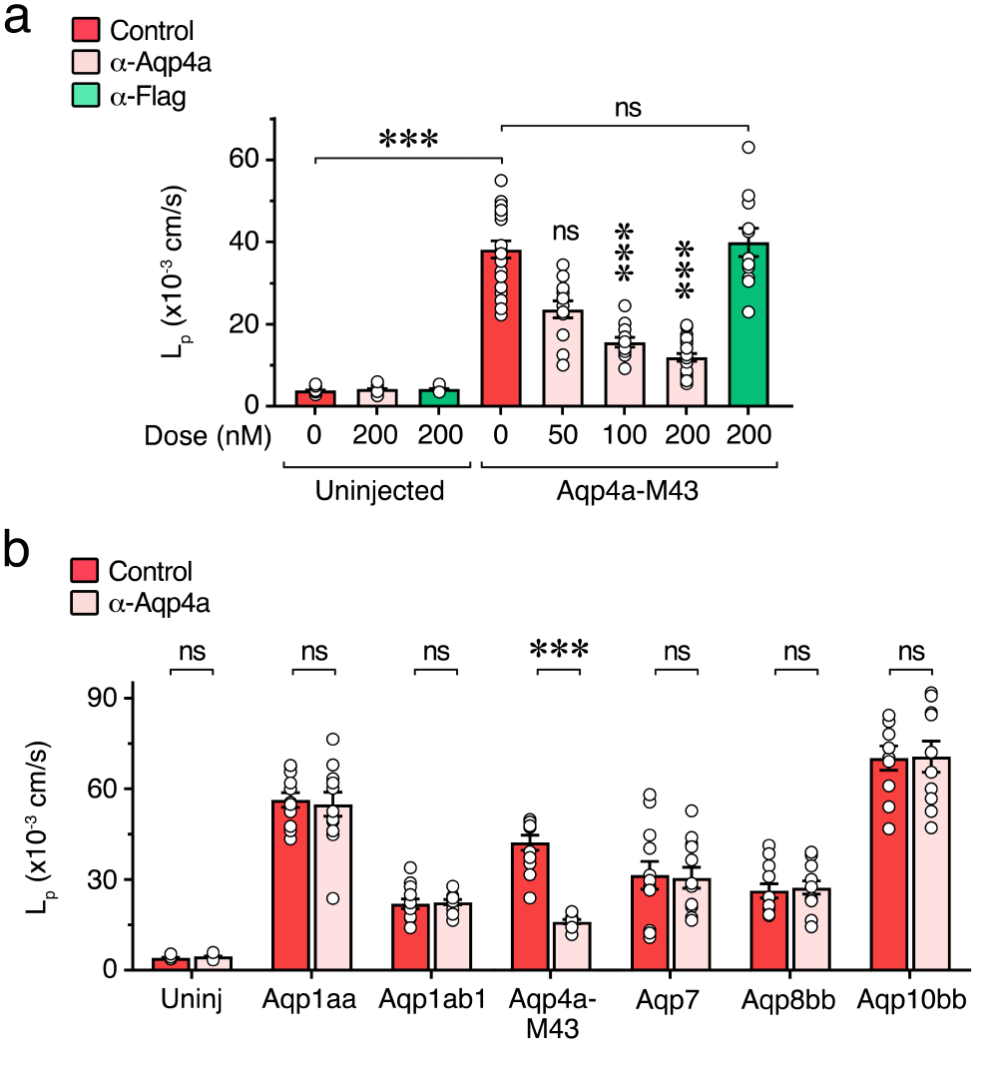
Specific immunological inhibition of seabream Aqp4a in *X. laevis* oocytes. **a** Water permeability (L_p_) of uninjected oocytes or oocytes expressing Aqp4a-M43 in the presence of 0.5% DMSO alone (red) or with increasing amounts of the seabream Aqp4a specific antibody (α-Aqp4a, pink). A chicken anti-Flag antibody (α-Flag) was used as negative control (green). **b** L_p_ of oocytes expressing distinct aquaporins in the absence (red) or presence of α-Aqp4a (200 nM, light red). The data are presented as the mean ± SEM (*n* = 10–24 oocytes/treatment; white dots) and were statistically analyzed by one-way ANOVA, or Kruskal-Wallis test, followed by Dunn’s multiple comparisons test (**a**), or by the Mann Whitney test (**b**). ***, *p* < 0.01 with respect to non-treated Aqp4a-expressing oocytes or as indicated in brackets; ns, not statistically significant

Finally, we tested the suitability of the anti-Aqp4a antibody (α-Aqp4a) to block Aqp4a-mediated water transport. For this, *X. laevis* oocytes heterologously expressing Aqp4a-M43 were exposed to increasing external concentrations of α-Aqp4a (50, 100 and 200 nM) for 1 h prior to L_p_ measurements under hypotonic conditions as above. The data showed that the L_p_ of Aqp4a-M43-expressing oocytes was inhibited by α-Aqp4a in a dose-response manner up to ~ 65% (with 200 nM), but unaffected by treatment with a chicken anti-Flag antibody (Fig. 7a). The effect of α-Aqp4a was specific since the permeability of oocytes expressing Aqp1aa, -1ab1, -7, -8bb or -10bb, was not affected by α-Aqp4a treatment (Fig, 7b). These results therefore indicate that exogenously added α-Aqp4a can specifically bind Aqp4a and inhibit channel-mediated water transport.

### Inhibition of Trpv4 and Aqp4a impairs sperm motility

**Fig. 8:**
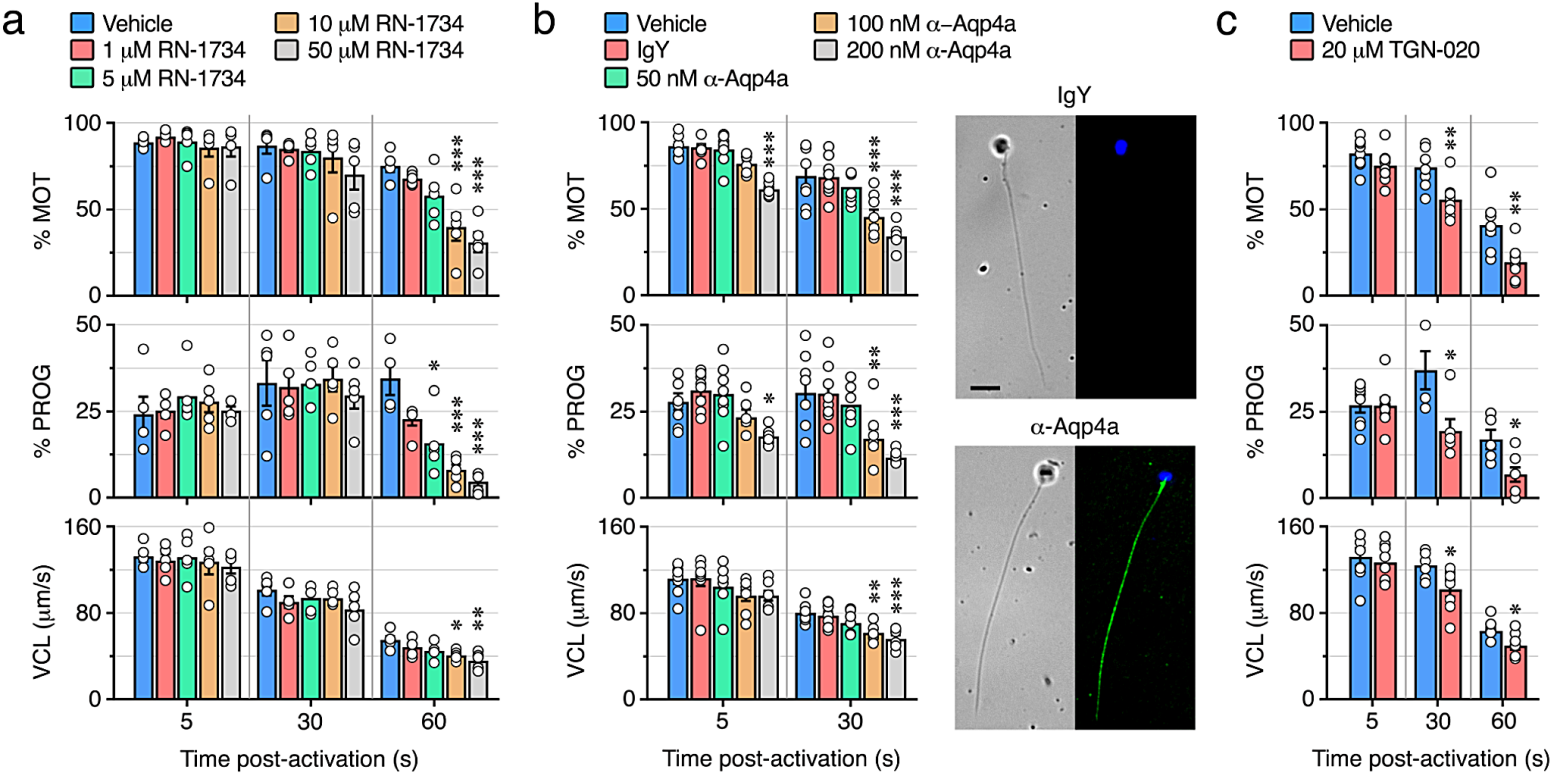
Inhibition of Trpv4 or Aqp4a impairs seabream sperm motility. **a-c** Inhibition of the percentage of motility and progressivity (% MOT and % PROG, respectively) and curvilinear velocity (VCL) at 5, 30 and 60 s post-activation induced by increasing doses of the TRPV4 antagonist RN-1734 or the Aqp4a antibody (α-Aqp4a), or by 20 µM of TGN-020. Control spermatozoa were treated with 0.5% DMSO (vehicle, **a** and **c**) or 200 nM IgY (**b**). In all panels, the data are the mean ± SEM (*n* = 5–8 males, one ejaculate per male; white dots). Statistical differences in **a** and **b** within each time point were measured by one-way ANOVA, or Kruskal-Wallis test, followed by Dunn’s multiple comparisons test (**a** and **b**), or by an unpaired Student *t*-test (**c**). *, *p* < 0.05; **, *p* < 0.01; ***, *p* < 0.001, with respect to non-treated sperm. The brightfield and immunostaining images in panel **b** confirm the specific binding of α-Aqp4a to Aqp4a in the spermatozoon flagellum through the labelling of either IgY- or α-Aqp4a in vitro-treated spermatozoa with anti-chicken secondary antibodies. The nucleus of the spermatozoa was counterstained with DAPI (blue). Scale bars, 5 μm

By using the above-characterized specific inhibitors, we next investigated the functional role of Trpv4 and Aqp4a during seabream sperm motility. In the first experiments, immotile spermatozoa were activated in seawater containing increasing doses of RN-1734 (1, 5, 10 or 50 µM), or 0.5% DMSO vehicle (controls), and the sperm kinetics determined at 5, 30 and 60 s post activation using CASA (Fig, 8a). None of the RN-1734 concentrations tested reduced the percentage of motile and progressive sperm (% MOT and % PROG, respectively), or the curvilinear velocity (VCL), at 5–30 s post activation. However, at 60 s clear inhibitory effects on the kinetic properties were observed with 10 and 50 µM of the antagonist, which reached > 50% for the % MOT and % PROG (Fig. 8a). These data suggest that the Trpv4_v1 channel, rather than the Trpv4_v10, is required for the post activation maintenance of sperm motility, but not for its initiation.

To test the effect of Aqp4a inhibition on motility, ejaculated sperm was preincubated in non-activating medium (NAM) containing increasing doses of α-Aqp4a (50, 100 or 200 nM), 200 nM IgY (control antibody), or 0.5% DMSO (control vehicle), for 1 h prior activation, and the sperm kinetics determined as above (Fig. 8b). Inhibitory effects of α-Aqp4a on the % MOT and % PROG of spermatozoa with respect to those treated with DMSO or IgY, were noted at 5 s post activation but only at the highest dose of the antibody, whereas the VCL was not affected. However, 100 and 200 nM of α-Aqp4a were effective at reducing the % MOT, % PROG and VCL at 30 s post activation (Fig. 8b). The specificity of Aqp4a immunological inhibition was confirmed by immunostaining of IgY- and α-Aqp4a-treated spermatozoa with a cyanine-3 (Cy3)-coupled anti-chicken IgY antibody, which labelled the flagella of spermatozoa treated with α-Aqp4a but not of those exposed to IgY (Fig. 8b, inset).

In subsequent experiments, immotile sperm was preincubated with 20 µM TGN-020 for 1 h, which reduced the %MOT, %PROG and VCL of spermatozoa at 30 s post activation (Fig. 8c), as seen with the α-Aqp4a. These observations suggest that, as for the Trpv4_v1, Aqp4a also plays a role in the maintenance of post-activated spermatozoon swimming performance, rather than in the activation of motility. However, the earlier detrimental effects of α-Aqp4a and TGN-020 with respect to those induced by RN-1734 during post activation indicate that Aqp4a may act upstream of the ion channel to sustain motility.

### Trpv4 and Aqp4a are involved in the RVI response in post-activated spermatozoa

**Fig. 9:**
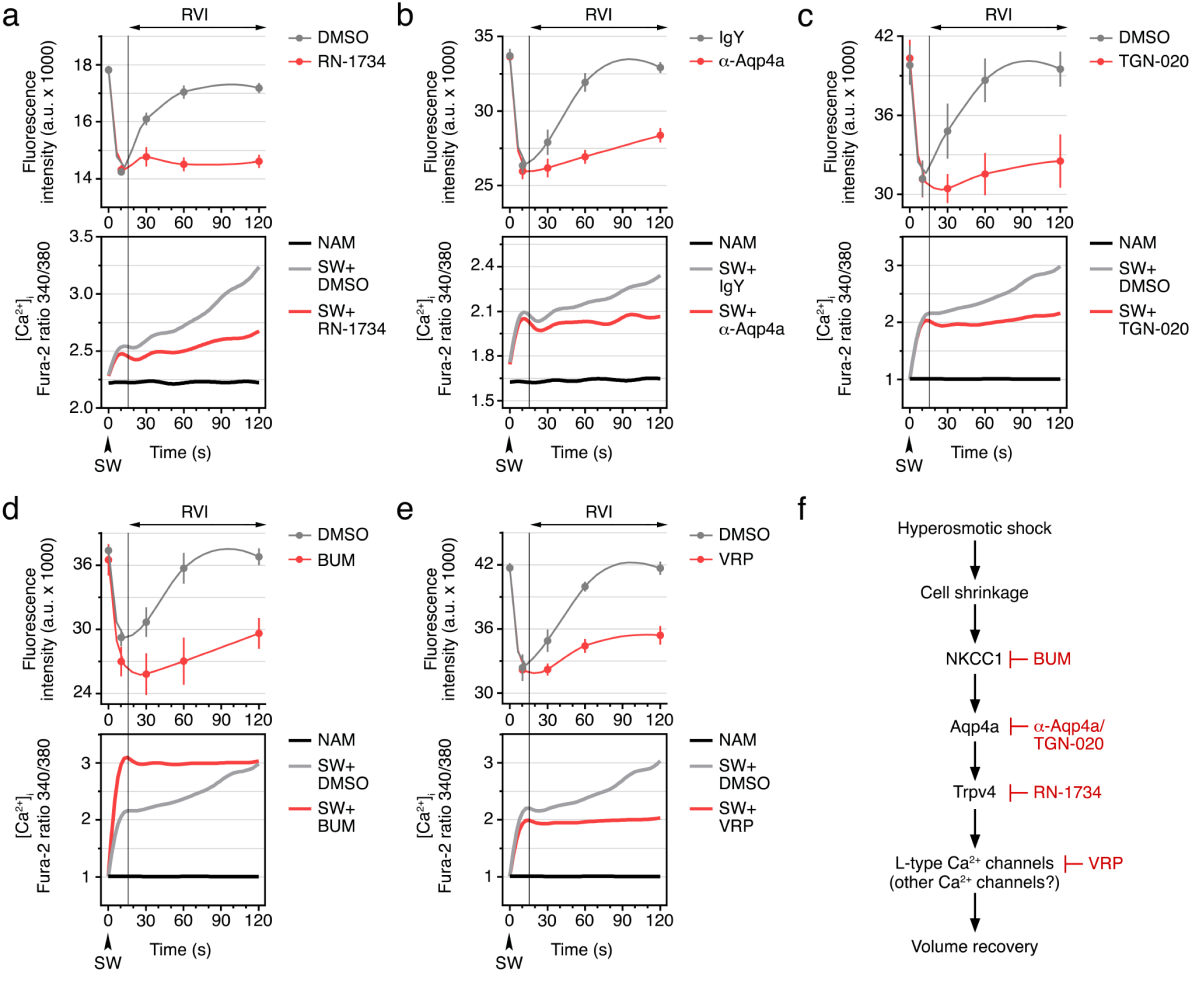
A complex network of Aqp4a and ion channels/transporters regulates the RVI response in post-activated seabream spermatozoa. Time-course changes in sperm volume and intracellular Ca^2+^ concentration ([Ca^+ 2^]_i_) before and after seawater (SW) activation in the presence of 50 µM RN-1734 (**a**), 200 nM of the α-Aqp4a (**b**), 20 μm TGN-020 (**c**), 50 µM of the NKCC1 inhibitor bumetanide (BUM, **d**) or 10 µM of the L-type Ca^2+^ channel blocker verapamil (VRP, **e**). The changes in [Ca^+ 2^]_i_ were also monitored in immotile spermatozoa maintained in non-activated medium (NAM, black lines). Control spermatozoa were exposed to 0.5% of the drug vehicle or IgY (200 nM) as appropriate for each experiment. In all panels, data are the mean ± SEM (*n* = 3–6 males, one ejaculate per male). **f** Proposed model for the sequential cascade of events allowing RVI in post-activated spermatozoa

Since both Trpv4_v1 and Aqp4a inhibition impaired sperm motility, we finally investigated whether this effect could be related to a defective RVI response. To test this hypothesis, we determined post activated volume and [Ca^2+^]_i_ changes in sperm treated with or without 50 µM RN-1734, 200 nM α-Aqp4a, or 20 µM TGN-020 (Fig. 9a-c). As previously observed, volumetric measurements using cell cytometry showed a rapid decrease in cell volume in control spermatozoa (treated with DMSO or IgY) within 10 s after seawater activation, and a subsequent RVI phase starting at ~ 15 s, with the initial sperm volume almost fully recovered at 120 s post activation (Fig. 9a-c, upper panels). Interestingly, in contrast to α-Aqp1aa, neither RN-1734, α-Aqp4a or TGN-020 affected the initial hypertonic-induced rapid cell shrinkage (Fig. 1d), thus confirming that Aqp1aa is the primary channel mediating this process. However, the three inhibitors prevented the RVI response (Fig. 9a-c, upper panels). Fluorometric quantification showed that [Ca^2+^]_i_ increased rapidly in controls and inhibitor-treated spermatozoa upon activation, according to the cell shrinkage occurring in all groups, but the further progressive increase of [Ca^2+^]_i_ in control spermatozoa was strongly reduced in the presence of RN-1734, or completely abolished with α-Aqp4a or TGN-020 (Fig. 9a-c, lower panels).

The above experiments suggest that Trpv4 and Aqp4a mediate the RVI response in post-activated seabream spermatozoa possibly through a Ca^2+^ influx. However, our previous results indicate that Trpv4 is activated only by cell swelling, which may therefore suggest that other ion channels and/or transporters upstream of Aqp4a and Trpv4 may be involved to allow the RVI process of sperm. A candidate mediator in this mechanism is the osmotic cell shrinkage-activated Na^+^/K^+^/2Cl^−^ cotransporter 1 (NKCC1) [45], the mRNA of which is detected in ejaculated seabream sperm [17]. To test this hypothesis, we employed the NKCC1 specific inhibitor Bumetanide (BUM) in activated spermatozoa undergoing RVI. In addition, in order to further investigate the role of the Ca^2+^ influx during volume recovery, we used the L-type Ca^2+^ channel blocker Verapamil (VRP), which reduces post-activated motility in seabream spermatozoa [17]. As for the other inhibitors, both BUM (50 µM) and VRP (10 µM) did not compromise cell shrinkage upon the hyperosmotic shock in seawater, but they almost fully prevented subsequent RVI (Fig. 9d and e, upper panels). Accordingly, BUM treatment impaired the kinematic properties of the spermatozoa (Supplementary Fig. S4), as previously shown for VRP [17]. The changes of [Ca^2+^]_i_ in BUM-treated sperm were however different from those observed with the previous inhibitors, since the Ca^2+^ levels in this group were higher than in the controls upon activation and did not increase further as in the untreated spermatozoa (Fig. 9d, lower panel). These unexpected observations might suggest an indirect increased Ca^2+^ release from intracellular stores, or the activation of Ca^2+^ channels, when NKCC1 is pharmacologically inhibited under hypertonic conditions. In contrast, VRP did not affect the initial [Ca^2+^]_i_ surge, but inhibited the progressive accumulation of Ca^2+^ seen in control sperm (Fig. 9e, lower panel). These data indicate that NKCC1 is required for the sperm RVI response, and confirm the role of L-type Ca^2+^ channels, and potentially other VRP-sensitive Ca^2+^ channels, in this mechanism, but not to initiate sperm motility as previously suggested [17].

Altogether our findings suggest that RVI in seabream sperm requires the sequential participation of Aqp4a and Trpv4 together with different ion channels and transporters. Our data suggest that the RVI response is initially mediated by NKCC1, which facilitates ion entry to osmotically drive water influx via Aqp4a causing spermatozoon swelling and further activation of the Trpv4 and L-type Ca^2+^ channels (Fig. 9f).

## Discussion

The present study demonstrates that marine piscine spermatozoa are able to perform an RVI response under high hypertonic stress, and provides novel insight into the molecular mechanisms underlying this mechanism. Immunolocalization and functional data suggest that the interplay between Aqp4a and Trpv4 channels, as well as of other ion channels and transporters, plays a critical role in the RVI response of marine spermatozoa.

The electrophysiological characterization of the three seabream Trpv4 splice variants investigated here suggests that the Trpv4_v1 isoform most closely reflects the functional ortholog of mammalian TRPV4 and freshwater fish Trpv4, which can be activated by cell swelling, and inactivated by cell shrinkage [24, 38, 46]. In mammals, the most distal region of the rat TRPV4 N-terminus is known to mediate the transduction of volume changes [38]. In the seabream, the N-termini of the Trpv4_v2 and _v10 isoforms are differentially spliced with respect to Trpv4_v1, and showed higher constitutive current activities without the ability to sense oocyte volume changes. These observations may suggest a conserved mechanism for the regulation of TRPV4-volume sensitivity in vertebrates, although the structural determinants in fish Trpv4 involved in volume sensing remain to be identified.

Immunofluorescence microscopy and immunoblotting revealed that seabream Aqp4a is localized along the flagellum of the ejaculated spermatozoon, thus extending the repertoire of functional aquaporins expressed in seabream sperm to seven paralogs, Aqp1aa, -1ab1, -4a, -7, -8bb and − 10bb [15], and Aqp3a (unpublished data). The higher expression of aquaporins in marine spermatozoa compared to mammalian sperm may reflect an adaptation to maintain flagellar motility and the swimming trajectory under a high hyperosmotic stress imposed by seawater. The expression of AQP4 orthologs in sperm may be unique to teleosts, since in mammals *AQP4* mRNA has only been detected in boar sperm after liquid storage [47]. In the plasma membrane of mammalian astrocytes, AQ4 isoforms (AQP4-M1, AQP4-M23, AQP4e, and AQP4ex) are able to aggregate in supramolecular assemblies termed orthogonal arrays of particles (OAP), in which AQP4 isoform composition may influence the RVD mechanism in response to cell swelling [37]. In seabream spermatozoa, Aqp4a seems to be expressed as two isoforms, Aqp4a-M1 and -M43, with higher predominance of the shorter Aqp4a-M43 isoform. Nevertheless, other Aqp4a-reactive bands were detected by immunoblotting, which may correspond to additional isoforms of the channel. The formation of Aqp4a OAPs in the membrane of seabream spermatozoa composed of different channel isoforms, and how these aggregates can affect RVI, remains to be investigated.

Using different cell volume measuring methods, our experiments show that TGN-020, a compound that has been proposed as a blocker of AQP4 in an ischemic cerebral edema disease model [48], efficiently blocks osmotic water permeation through seabream Aqp4a expressed in *X. laevis* oocytes. These observations are consistent with previous findings for human, mouse and rat AQP4 [29]. The effect of TGN-020 is considered to be mediated by its direct binding to specific residues at the entrance of the AQP4 pore thereby blocking water conductance [42]. Based on the present observations that the putative TGN-020 binding residues are highly conserved in the fish ortholog, this is likely the mechanism by which TGN-020 reduces seabream Aqp4a-mediated water permeability. As in mammals, the effect of TGN-020 was selective towards Aqp4a, since the compound did not block any of the other tested aquaporins expressed in the seabream spermatozoa. These data therefore validate TGN-020 as a suitable Aqp4a blocker for the pharmacological inhibition of Aqp4a in seabream spermatozoa. However, in contrast to mammalian AQP4, we observed that the Aqp4a-M1 isoform, bearing a much longer N-terminus than the M43 isoform, was apparently insensitive to TGN-020, despite the apparent spatial conservation of the binding residues in both isoforms and their equal targeting to the plasma membrane of oocytes. The structural features of Aqp4a-M1 preventing TGN-020 inhibition of the channel are unknown, although it is possible that conformational changes due to the unusually long N-terminus may be implicated.

In ejaculated seabream spermatozoa, Trpv4 and Aqp4a co-localize along the flagellum and are readily co-immunoprecipitated, and thus seem to form a molecular complex, as reported for AQP4 and TRPV4 in astrocytes, salivary gland and retinal Müller cells [23, 27, 28]. The pharmacological and immunological inhibition of Aqp4a, as well as antagonist-mediated blockage of Trpv4, did not affect the initial decrease in sperm volume upon release into seawater and the activation of motility, but prevented or highly reduced the progressive influx of Ca^2+^ and the RVI response in the post-activated spermatozoon. Inhibition of either Aqp4a or Trpv4 ultimately impaired the sperm motility kinetics, suggesting that the Trpv4/Aqp4a signaling complex may be a mechanism to respond to hyperosmotic stress to maintain the swimming performance of seabream spermatozoa. These data confirm previous observations [16], indicating that the initial cell shrinkage necessary to trigger sperm motility is mediated by rapid water efflux through Aqp1aa. The plausible absence of Aqp4a-mediated water fluxes within the first 5 s post activation suggest that this channel is not active during this time frame, either because its trafficking to the spermatozoon plasma membrane is not promoted [49, 50] and/or because its function is controlled by gating mechanisms [51–54]. Similar mechanisms might also regulate the function of Aqp1aa at post activation to allow RVI in post-activated spermatozoa.

**Fig. 10:**
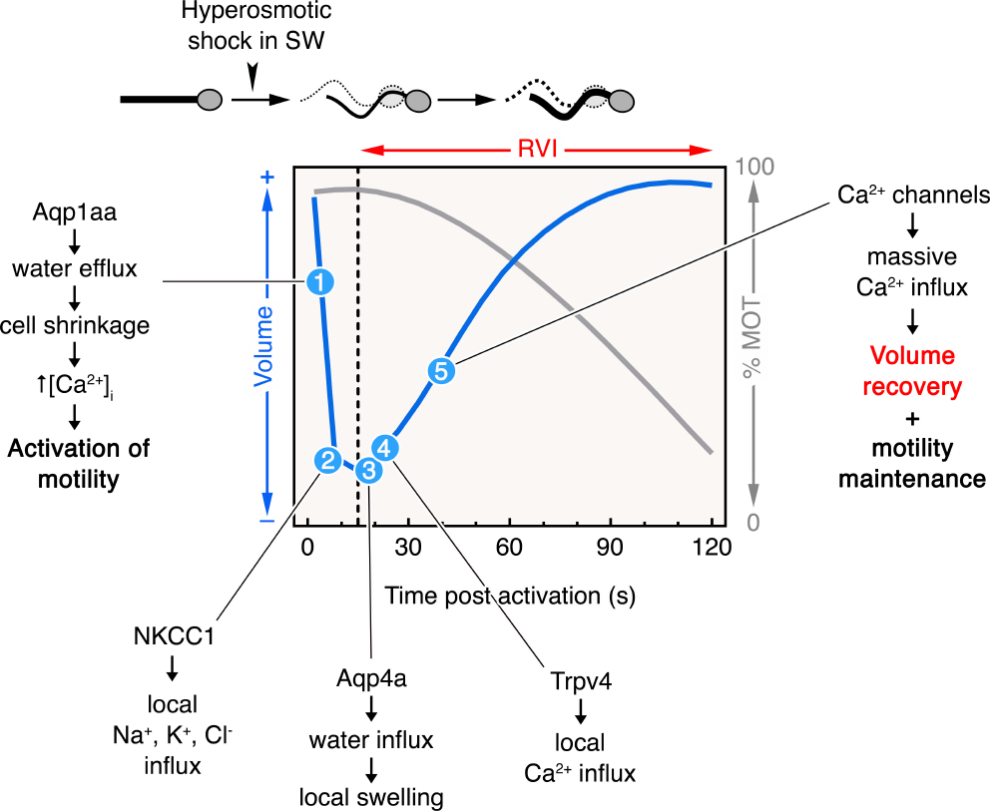
Model for the RVI mechanism in post-activated seabream spermatozoa. Upon release into the hyperosmotic seawater a rapid water efflux mediated by Aqp1aa results in cell shrinkage and subsequent [Ca^2+^]_i_ increase, both triggering flagellar motility. The rapid spermatozoon shrinkage could induce the activation of NKCC1 and Na^+^, K^+^, Cl^−^ influx, which would then drive water uptake through Aqp4a, and perhaps also by NKCC1 supported water fluxes. Water influx into the spermatozoon could induce local swelling of the flagellum, triggering the activation of the Trpv4 and a further local Ca^2+^ influx, which may activate signaling events for the stimulation of L-type Ca^2+^ channels, and perhaps of other Ca^2+^ channels present in the spermatozoa, thereby allowing a massive Ca^2+^ influx. The increased accumulation of Ca^2+^ and other ions would facilitate further Aqp4a-mediated water uptake in the spermatozoon, and feasibly also through Aqp1ab1 and/or -10bb present in the anterior region of the flagellum, to promote a fast volume recovery and to maintain motility

The remarkable observation that Trpv4, which is typically activated by cell swelling to mediate an RVD response is in fact required for RVI in activated marine spermatozoa under high hypertonic stress, prompted us to investigate the potential role of other ion channels or transporters upstream of the Trpv4-mediated RVI mechanism. Our data show that the cell shrinkage-activated NKCC1 is one of these mechanisms since its inhibition greatly reduced the RVI response. These observations are consistent with studies in boar spermatozoa, in which protein tyrosine kinase-dependent pathway and Na^+^ and Cl^−^ fluxes are suggested to regulate RVI after exposure to mild hypertonic conditions [55]. NKCC1 is also essential for mouse spermatogenesis [56] and sperm capacitation [57]. Based on these findings, we propose a model to explain the mechanism of RVI in seabream spermatozoa under strong hypertonic conditions (Fig. 10). Upon release into the hyperosmotic seawater a rapid water efflux mediated by Aqp1aa results in cell shrinkage and subsequent [Ca^2+^]_i_ increase, both triggering flagellar motility. The rapid spermatozoon shrinkage could induce the activation of NKCC1 and Na^+^, K^+^, Cl^−^ influx, which would then drive water uptake through Aqp4a, and perhaps also by NKCC1 supported water fluxes [58, 59]. Water influx into the spermatozoon promotes local swelling of the flagellum, triggering the volume sensing activation of the Trpv4 and a further local Ca^2+^ influx, which may activate signaling events for the stimulation of L-type Ca^2+^ channels, and perhaps of other Ca^2+^ channels present in the spermatozoa [17], to generate the observed massive Ca^2+^ influx. The increased accumulation of Ca^2+^ and other ions would further facilitate Aqp4a-mediated water uptake in the spermatozoon, and perhaps also through Aqp1ab1 and/or -10bb present in the anterior region of the flagellum [16], to promote the fast volume recovery. This model suggests the interplay of water channels, volume sensing and ion channels/transporters to provoke the RVI response in post-activated marine spermatozoa, each of which is supported by our data. However, additional experimentation is necessary to elucidate the molecular mechanisms regulating the function of these channels, and to identify additional players involved in RVI.

In summary, the present work shows that both Aqp4a and Trpv4 co-operatively regulate an RVI response in post-activated seabream spermatozoa under high hypertonic stress, which is essential for motility maintenance. However, this mechanism appears to be complex and requires the interplay with other ion channels and transporters to further explain the ion flux dynamics. The findings nevertheless reveal that an adaptive integrated network of water, volume sensing and ion channels evolved in marine fish to cope with the high hyperosmotic stress imposed by seawater to maintain spermatozoon swimming performance and fertilization competence.

### Methods

#### Animals

Adult 2-3-year old gilthead seabream were maintained at the facilities of the Institut de Ciències del Mar (CSIC, Barcelona) as previously described [15]. Samples of ejaculated sperm were obtained from males during the natural reproductive season (November-February). Fish were sedated by immersion in seawater with 500 ppm of 2-phenoxyethanol, and ejaculated sperm was collected after a soft pressure to the abdominal area of the fish using a syringe located in the gonopore to avoid seawater or urine contamination. Adult *X. laevis* were purchased from the Centre de Ressources Biologiques Xénopes (University of Rennes, France) and maintained at the AQUAB facilities of the Universitat Autònoma de Barcelona (UAB, Spain). Frogs were kept in tanks with filtrated freshwater at 18ºC, under a 12-h light-dark cycle, and fed two days a week with beef heart or pellets (*Xenopus* Sticks, AQUA Schwarz GmbH, Göttingen, Germany). Oocytes were collected by surgical laparotomy from anesthetized females. In some experiments, oocytes were purchased from Ecocyte Bioscience (Germany). Procedures relating to the care and use of fish and sample collection, and for surgical laparotomy of female frogs, were approved by the Ethics Committee of CSIC, the Ethics Committee for Animal and Human Experimentation from UAB, and the Catalan Government (Direcció General de Polítiques Ambientals i Medi Natural; Projects nos. 10,985 and 12,147).

### Antibodies and chemicals

Affinity-purified chicken polyclonal antibody specific for seabream Aqp4a has been described elsewhere [35]. The HA tag rabbit antibody was from Thermo Fisher Scientific (Invitrogen, # PA1-985), whereas the chicken anti-Flag antibody was from Gallus Immunotech Inc. (# AFLAG). Antibodies against human or mouse TRPV4 were purchased from Novus Biologicals (# NBP2-41262) and Invitrogen (# OSR00136W), respectively. All other reagents were purchased from Merck unless indicated otherwise.

### Sequence, structure and phylogenetic analyses

The phylogenetic analysis was conducted via Bayesian inference (MrBayes v3.2.2) as described previously [60] using 1 million MCMC generations (aamodel = mixed) of a ClustalX alignment of 27 vertebrate Trpv4 orthologs. Sequences used are listed in Supplementary Table S1, and the amino acid alignment is shown in Supplementary Data S1. The three-dimensional model of human AQP4 was obtained from the protein data bank (PDB: 3gd8), and the seabream Aqp4a-M1 and -M43 variants modeled via the automatic SWISS-MODEL pipeline [61] using the A0A4U5UWT1 and 2zz9 templates, respectively. Each model was rendered with PyMOL (pymol.org).

### Isolation of seabream Trpv4 cDNAs

Full-length cDNA encoding seabream Trpv4_v1 was isolated by RT-PCR using testis total RNA as previously described [35]. Oligonucleotide primers bearing *EcoR*V and *Spe*I restriction sites were designed based on seabream genome sequence data available in GenBank (accession no. XM_030415727). The forward and reverse primers were 5’-*gatatc*TGCTTCAGCTTCATTTCCAG-3’ and 5-*actagt*TCGTCCCCAGCAGAGTTATC-3, respectively. The full-length cDNA sequences of the Trpv4_v2 and _v10 variants were synthesized (Life Technologies) following the Ensembl (version fSpaAur1.1; https://www.ensembl.org/Sparus_aurata/Info/Index) transcript IDs ENSSAUT00010061488.1 and ENSSAUT00010061519.1, respectively.

### Heterologous expression in *X. laevis oocytes*

Heterologous expression of the different Trpv4 cDNAs, as well as of the seabream Aqp4a-M1 or -M43 (GenBank accession no. KY682700), was carried out as described previously [24, 62]. The cDNAs were subcloned into the oocyte expression vector pT7Ts and in vitro transcribed using T7 mMessage machine according to manufacturer’s instructions (Ambion, #AM1344). The cRNAs were extracted with MEGAclear (Ambion, #AM1908). The cRNAs were injected into oocytes in different amounts: 0.5 ng for Aqp1aa, 1 ng for Aqp1ab1, 5 ng for Aqp4a-M1, Aqp4a-M43, Aqp8bb, Trpv4_v1, Trpv4_v2 and Trpv4_v10, and 10 ng for Aqp7 and Aqp10bb. Control oocytes were not injected. For electrophysiological recordings, the oocytes were kept in Kulori medium (90 mM NaCl, 1 mM KCl, 1 mM CaCl_2_, 1 mM MgCl_2_, 5 mM HEPES, pH 7.4) containing 100 µM ruthenium red (RR, Merck #R-2751) to suppress basal Trpv4 activity [63] for 3–4 days at 18˚C prior to the experiments. In other experiments, the oocytes were kept in Modified Barth’s Solution (MBS) (88 mM NaCl, 1 mM KCl, 2.4 mM NaHCO_3_, 0.82 mM MgSO_4_, 0.33 mM Ca(NO_3_)_2_, 0.41 mM CaCl_2_, 10 mM HEPES, 25 mg/ml gentamycin, pH 7.5) for 3 days at 18ºC.

### Electrophysiology and volume measurements of oocytes

Conventional two-electrode voltage clamp studies were performed with a DAGAN CA-1B High Performance oocyte clamp (DAGAN, Minneapolis, MN) with Digidata 1440 A interface controlled by pCLAMP software, version 10.5 (Molecular Devices, Burlingame, CA). Electrodes were pulled (HEKA, PIP5) from borosilicate glass capillaries to a resistance of 2–4 MΩ when filled with 1 M KCl. The current traces were obtained by stepping the clamp potential from − 20 mV to test potentials ranging from + 50 mV to -130 mV (200-ms pulses) in increments of 15 mV. Recordings were low pass-filtered at 500 Hz and sampled at 1 kHz. Oocytes expressing the seabream Trpv4 variants, Aqp4a or both, were placed in an experimental chamber, perfused and volume measurements were performed by sequential image analysis as described previously [64]. The perfusion solution consisted of 50 mM NaCl, 2 mM KCl, 1 mM MgCl_2_, 1 mM CaCl_2_, 10 mM HEPES, 100 mM mannitol (Tris buffered pH 7.4, 220 mOsm/L). A hypotonic solution was made by the removal of 100 mM mannitol with resulting a osmolarity of 120 mOsm/L, and a hypertonic solution was made by addition of 100 mM mannitol (320 mOsm/L). Osmolarities of all solutions were confirmed to an accuracy of ± 1 mOsmol with an osmometer Type 15 (Löser Messtechnik, Berlin, Germany). In experiments where different TRPV4 antagonists were tested, oocytes expressing Trpv4_v1 or _v10 alone were maintained in isosmotic solution (220 mOsm) and inhibitors were applied through the perfusion system. The RR and HC-067047 (Merck, # SML0143) inhibitors were tested at 1 and 10 µM, whereas the RN-1734 (Merck, # 616,520) inhibitor was used at 10 and 100 µM. Both RN-1734 and HC-067047 were dissolved in DMSO, while the RR was dissolved in water. Because variability in Trpv4-mediated basal currents was observed, all experiments were set up as pair-wise trials, except for those in which we tested the effect of 1 h-incubation with the TRPV4 antagonists. The electrophysiological recordings were carried out at room temperature.

### Oocyte swelling assays

Volume recordings of oocytes expressing Aqp4a were carried out as above and the L_p_ of the oocytes was determined following an abrupt introduction of a hyposmotic challenge (-100 mOsm or -200 mOsm) during sequential recording of the oocyte volume changes by image analysis as described previously [29]. Hyposmotic solutions were made by the removal of mannitol from the perfusion solution or MBS, and osmolarities of all solutions were verified with an osmometer. Volume changes in the presence or absence of TGN-020 were recorded after 5 min of treatment with 20 µM of the inhibitor with the oocytes serving as own controls. In experiments with oocytes expressing different seabream aquaporin paralogs, oocytes were treated with TGN-020 in isotonic MBS (220 mOsm) [62] for 1 h with shaking at room temperature, and subsequently directly exposed to 20 mOsm MBS for swelling recordings in a non-paired experimental design. The same type of experiments was carried out for the immunological inhibition of Aqp4a-mediated water transport, which was assessed by preincubating uninjected and injected oocytes with increasing concentrations of the seabream α-Aqp4a (50, 100 or 200 nM) or 200 nM of chicken anti-Flag, in the presence of 0.5% DMSO, for 1 h at 18ºC prior to the swelling assays. In all experiments, control oocytes were exposed to 0.5% DMSO alone.

### RT-PCR

Total RNA from testis and ejaculated spermatozoa was extracted using the Qiagen RNeasy Plus Minit Kit following the manufacturer’s instructions. The cDNA was synthesized from 76 ng to 1 µg of total RNA using the AccuScript High-Fidelity 1st Strand cDNA Synthesis Kit (Agilent, # 200,820). The PCR reaction was performed using 1 µl of cDNA, 5 IU Taq DNA polymerase, and 0.2 M of forward and reverse primers specific for the selected genes (Supplementary Table S2). Reactions were amplified using an initial denaturing step for 2 min at 94 °C, followed by 35 cycles of 94 °C for 1 min, 60 °C for 1 min, and 72 °C for 2 min, ending with a final elongation at 72 °C for 7 min. PCR products were run on 1% agarose gels and photographed.

### Protein extraction and immunoblotting

The total membrane fraction of *X. laevis* oocytes was isolated as described previously [65]. For protein extraction from whole spermatozoa, fresh samples of ejaculated sperm were diluted in NAM (10^9^ cells/ml) and subsequently mixed with ice-cold 2 x RIPA buffer containing 300 mM NaCl, 100 mM Tris-HCl, pH 8, 2% Triton X-100, 1% sodium deoxycholate, 2 mM EDTA, 2 mM EGTA, 2 mM Na_3_VO_4_, 2 mM NaF, 200 U of benzonase (Merck, # 103,773) and EDTA-free protease inhibitors (Merck, # 11,836,170,001). Cells were dissociated with a glass dounce homogenizer, sonicated for 20 s at 20% amplitude using a Digital Sonifier^®^ S250D (Branson), and centrifuged at 14,000 × *g* for 10 min at 4 °C. The supernatant was mixed with 2 × Laemmli sample buffer containing 5% β-mercaptoethanol, heated at 95 °C for 15 min, deep frozen in liquid nitrogen, and stored at -80 °C. For the isolation of the sperm flagella, NAM-diluted ejaculated spermatozoa (3 × 10^9^ cells) were subjected to mechanical separation of head and flagellum as described previously [15]. In some experiments, protein extract (50 µg) was deglycosylated by incubation with 500 units of PNGase F (New England Biolabs Inc., # P0704S) for 3 h at 37ºC prior to SDS-PAGE.

For immunoblotting, protein extracts were denatured at 95 °C for 10 min, electrophoresed in 7–12% SDS-PAGE and blotted onto Immun-Blot nitrocellulose 0.2 μm membranes (Bio-Rad Laboratories, Inc.), as described previously [15]. The membranes were blocked with either 5% non-fat dry milk or 3% bovine serum albumin (BSA) diluted in TBST (20 mM Tris, 140 mM NaCl, 0.1% Tween, pH 7.6), and incubated overnight at 4ºC, with the Aqp4a or TRPV4 antibodies (1:500 dilution). Bound antibodies were detected with horseradish peroxidase-coupled anti-chicken IgY or anti-rabbit IgG antibodies for 1 h at room temperature. After washing in TBST, immunoreactive bands were revealed by the Immobilon™ Western chemiluminescent HRP substrate (Merck, #WBKLS). The specificity of the anti-Aqp4a antibody in spermatozoa was assessed by preabsorbing the antibody with its corresponding peptide (1:5 ratio) for 1 h at 37ºC prior to dilution in milk and incubation with the membrane.

### Co-immunoprecipitation

Sperm cells (10^9^) were lysed in 1 ml of immunoprecipitation RIPA buffer (150 mM NaCl, 50 mM Tris pH 8.0, 1.0% Triton X-100, 0.5% sodium deoxycholate, 0.1% SDS, supplemented with 1 mM NaF, 1 mM Na_3_VO_4_, protease inhibitors, and 80 U benzonase) and centrifuged at 13,000 × g for 10 min at 4ºC. An aliquot (10%) of the supernatant was collected as “input” and mixed with 2 × Laemmli sample buffer with protease inhibitors. The remaining supernatant was mixed with activated G-protein beads (Pure Proteome™ Protein G Magnetic Beads) previously coupled to TRPV4 antibodies or rabbit IgG as negative control following the manufacturer’s instructions, and incubated overnight at 4 °C under constant agitation. The beads were further washed three times with PBS (137 mM NaCl, 2.7 mM KCl, 10 mM Na_2_HPO_4_, 1.8 mM KH_2_PO_4_, pH 7.5) with 0.05% Tween-20 (PBST), and eluted in 50 µl of 1 × Laemmli sample buffer supplemented with protease and phosphatase inhibitors. The input and immunoprecipitated samples were immunoblotted as indicated above.

### Immunofluorescence microscopy

*X. laevis* oocytes expressing Aqp4a and HA-tagged Trpv4_v1, _v2 or _v10 were fixed for 6 h in 4% paraformaldehyde (PFA) in PBS, dehydrated, and mounted in paraffin as described elsewhere [66]. Section (8 μm) were blocked with PBST containing 5% normal goat serum and 0.1% BSA for 1 h at room temperature. Double immunostaining of Aqp4a and Trpv4 variants was carried out using the HA rabbit antibody and the chicken seabream Aqp4a antiserum (1:400 dilution) overnight at 4ºC in a humidified chamber. After washing three times with PBS, slides were incubated with sheep anti-rabbit IgG coupled with Cy3 (Merck, # C2306,) and goat anti-chicken IgY coupled with Alexa Fluor 488 (Invitrogen, # A-11,039), both at 1:800 dilution, for 1 h at room temperature. Sections were washed with PBS and incubated (1:10000) with WGA Alexa Fluor^®^ 647 conjugate (Life Technologies Corp., # W32466) for 10 min, and subsequently with 4′,6-diamidino-2-phenylindole (DAPI, Merck, # G8294, 1:5000) for 3 min. The sections were mounted with fluoromount aqueous anti-fading medium (Merck, # F4680), and images were acquired at 100 × magnification with a Zeiss Axio Imager Z1/ApoTome fluorescence microscope (CarlZeiss Corp., Oberkochen, Germany).

Spermatozoa were attached to UltraStick/UltraFrost Adhesion slides (Electron Microscopy Sciences, Hatfield, PA, USA) and directly fixed on the slide in 4% PFA in PBS for 15 min. Slides were rinsed in PBS and permeabilized with boiling citrate at 0.01 M and pH 6 for 5 min, repeated 3 times. After the citrate solution was cooled down at room temperature, the slides were washed in PBS and subjected to a second permeabilization step in 1 M HCl for 20 min at 37˚C. Slides were rinsed again in PBS for 10 min. and subsequently incubated with 0.2% Triton X-100 for 15 min at room temperature. Blocking and incubation with primary and secondary antibodies were conducted as above. The cells were counterstained with DAPI (1:5000) for 3 min in PBS to stain the nuclei, and the slides were mounted and photographed as described above.

### Sperm motility assays

The kinematic parameters of seabream sperm were determined by CASA using the Integrated Semen Analysis System (ISAS^®^v1, Proiser) software as previously described [15, 17]. Sperm (10^8^ cells/ml diluted in NAM) was activated in the absence or presence of increasing concentrations of RN-1734 (1–50 µM) or α-Aqp4a (50–200 nM), 20 µM of TGN-020, 50 µM of BUM or 10 µM VRP. Except for RN-1734, NAM-diluted sperm was preincubated with the inhibitors for 15 min (BUM and VRP) or 1 h at 16ºC (α-Aqp4a and TGN-020) prior to activation in seawater. Control sperm was treated with 0.5% DMSO with or without 200 nM IgY. In some experiments, sperm was activated in 1.1 M sucrose. The sperm motility parameters were recorded in triplicate for each ejaculate.

#### Determination of sperm volume and [Ca^2+^]_i_

Seawater-activated sperm treated with the different inhibitors, as well as with 10 µM of VRP, were collected 0, 10, 30, 60–120 s post activation and immediately fixed with 4% PFA. After washing in PBS, sperm was diluted to 10^7^ cells/ml in PBS and incubated with 1 µM of Alexa Fluor™ 488-coupled WGA. The intensity of fluorescence was analyzed within 1 h with a previously calibrated Cytoflex cytometer (Beckman Coulter Life Sciences) and acquired and analyzed using the CytExpert software (Beckman Coulter Life Sciences), following the methods described by Kekäläinen et al. [67]. After stabilization of the stream for 20 s, the samples were run at low flow rate (ca. 10 µL/min) with PBS as sheath fluid. Samples were excited with a 488 nm air-cooled solid state 20 mW laser, and green fluorescent emission was detected with the 530/30 bandpass filter. Enumerations of 10,000 counted cells per sample were recorded on the basis of their green fluorescence, once the background (autofluorescence of sperm cells without WGA staining) was established, and the corresponding threshold was applied to green fluorescence. Cells were detected in a plot of FITC-A fluorescence intensity versus forward scatter (FSC: particle size, width). Results were presented as the mean values of FITC fluorescence intensity determined in triplicate for each time point following seawater activation. Part of the fixed activated sperm used for cytometry at time 0, 10 s and 120 s was attached on UltraStick/UltraFrost Adhesion slides and counterstained with DAPI as above before imaging on a Zeiss LSM 980 Airyscan 2 Confocal with the PicoQuant FLIM module (Zeiss corp.).

Intracellular Ca^2+^ levels in sperm were estimated using the Fura-2-AM cell permeant dye (Invitrogen, #F1221) as described previously [19]. Measurements were taken in duplicate or triplicate for each ejaculate.

### Statistical analysis

The statistical significance among multiple groups was analyzed by one-way ANOVA, or the nonparametric Kruskal-Wallis test, followed by the Dunnett’s multiple comparison test. Comparisons between two independent groups were made by the two-tailed unpaired Student’s *t*-test, or by the nonparametric Mann Whitney test when variances were not equal, or by the one sample *t*-test when analyzing normalized currents. Percentages were square root transformed prior to analyses. Statistical analyses were performed using GraphPad Prism v9.4.1 (681) software (GraphPad Software). In all cases, statistical significance was defined as *p* < 0.05 (*), *p* < 0.01 (**), or *p* < 0.001 (***).

### Electronic supplementary material

Below is the link to the electronic supplementary material.

Supplementary Material 1

Supplementary Material 2

Supplementary Material 3

Supplementary Material 4

## Data Availability

Data from this study, including raw data tables, alignments, and uncropped gels and blots, are available in the Supplementary material.
