# Supplementary material for: Aqp4a and Trpv4 mediate regulatory cell volume increase for swimming maintenance of marine fish spermatozoa
Source: Cell Mol Life Sci. 2024 Jul 6;81(1):285. doi: 10.1007/s00018-024-05341-w (PMC11335209; doi:10.1007/s00018-024-05341-w)
Supplement: Supplementary file 1 — Supplementary Material 1 [file 18_2024_5341_MOESM1_ESM.pdf]

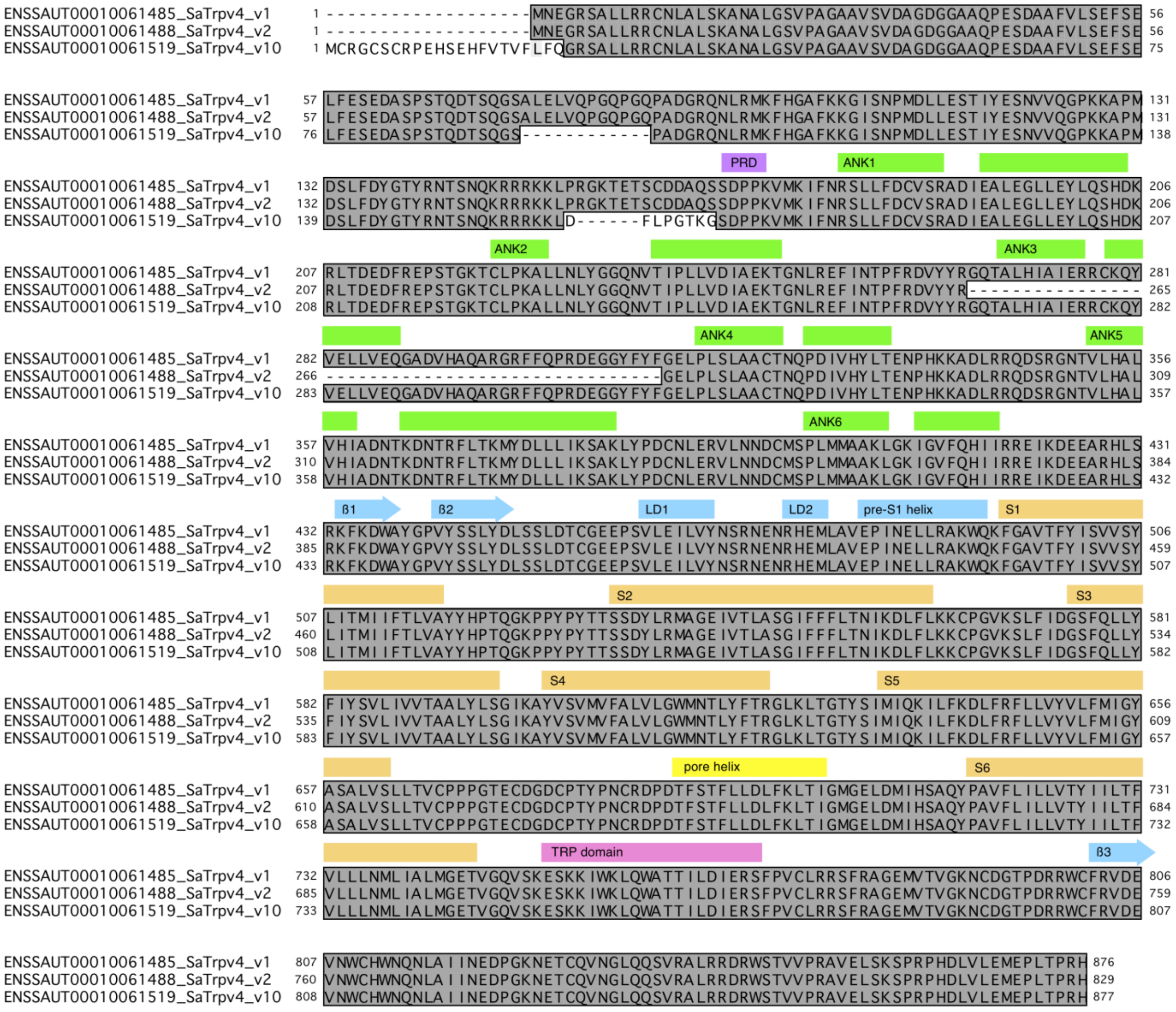

Figure S1. Multiple sequence alignment (Clustal X) of the gilthead seabream (*Sparus aurata*) Trpv4 isoforms, Trpv4_v1, Trpv4_v2 and Trpv4_v10, with Ensembl (v109) accession numbers. Structural features including the proline rich domain (PRD), ankyrin repeats (ANK1-6), beta sheets (ß1-3), linker domains (LD1-2), pre S1 helix, membrane-spanning regions (S1-6), pore helix, and the transient receptor potential (TRP) domain are annotated after Deng et al. (2018).

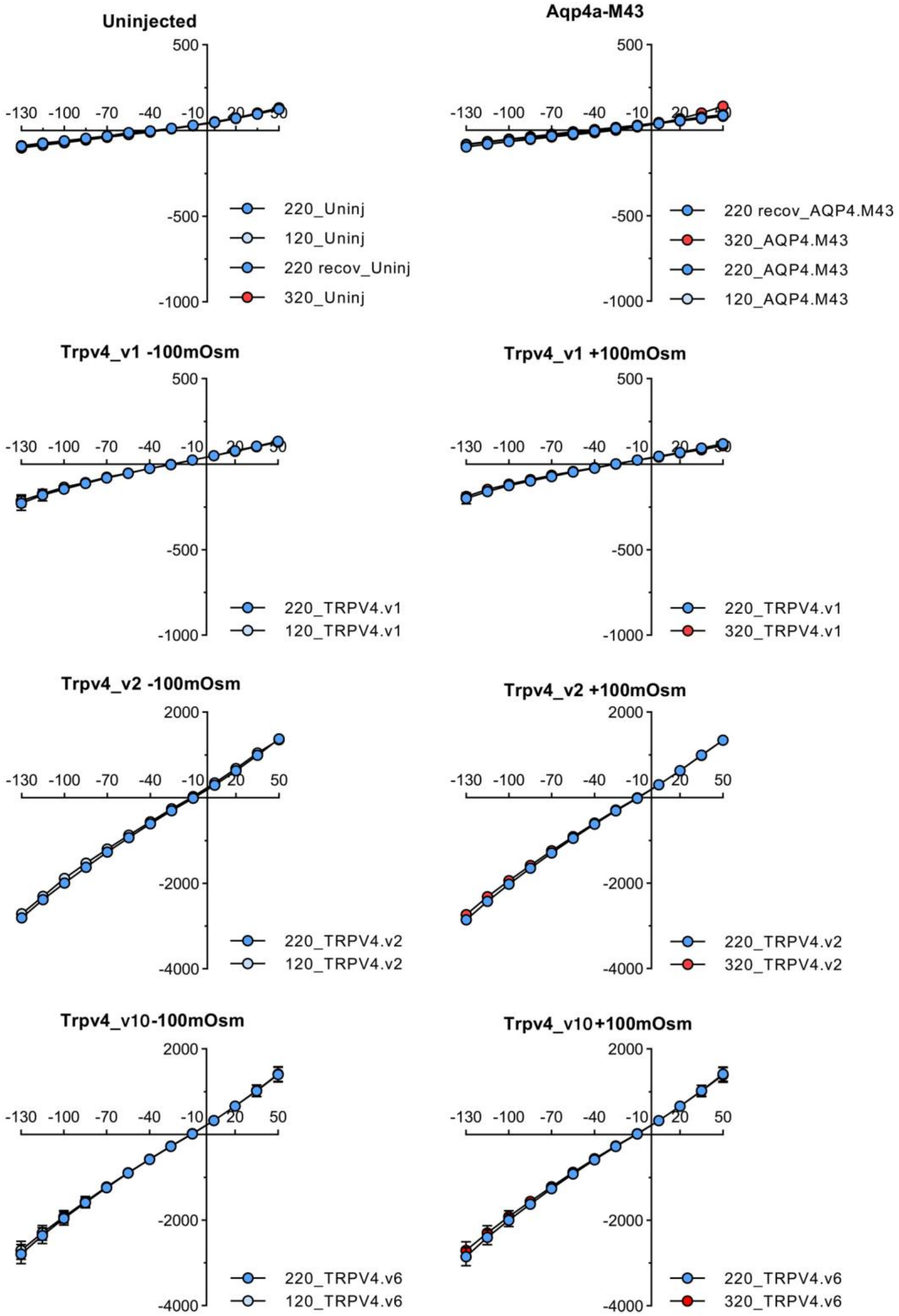

Figure S2. Controls from Figure 2. Summarized I/V curves from *X. laevis* uninjected oocytes and oocytes expressing Aqp4a or Tpv4_v1, Trpv4_v2 or Trpv4_v10 in isosmotic control solution (white) or during application of a hyposmotic (blue) or hyperosmotic solution (red).

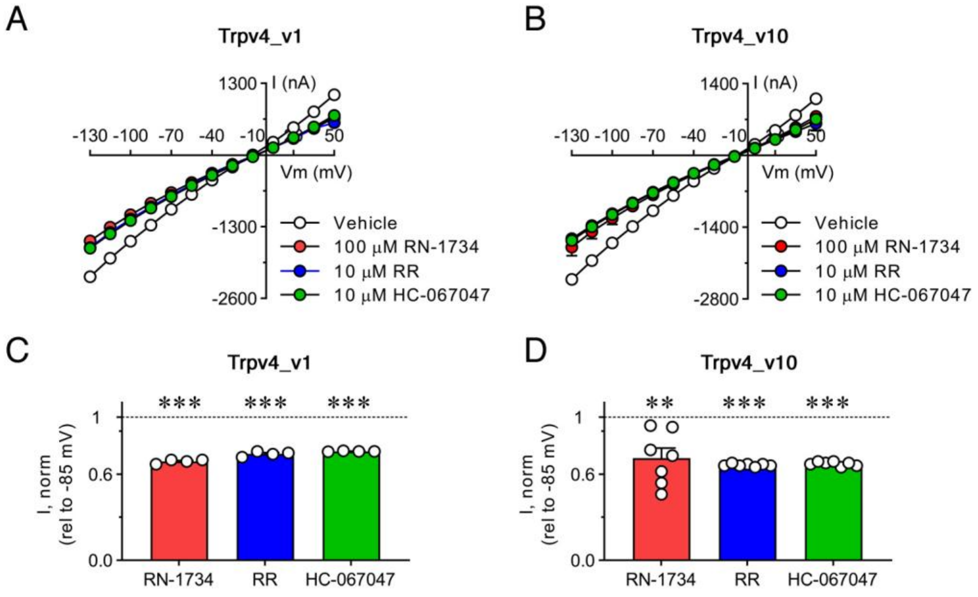

Figure S3. Inhibition of Trpv4_v1 and Trpv4_v10 after long-term exposure to different blockers in *X. laevis* oocytes. (***A*** and ***B***) Summarized I/V curves from oocytes expressing Trpv4_v1 or Trpv4_v10 in control solution (white) or after incubation with the TRPV4 blockers RN-1734 (100 µM, red), ruthenium red (RR) (10 µM, blue) and HC-067047 (10 µM, green) for 1 h. (***C*** and ***D***) Trpv4_v1 or Trpv4_v10-mediated current activity at -85 mV obtained after exposure to the different TRPV4 blockers was normalized to that obtained in control conditions. The paired normalized values (*n* = 5-8 oocytes, white dots) are presented as mean ± SEM, and were statistically analyzed by one sample *t*-test (**, *p*< 0.01; ***, *p* < 0.001; with respect to the same oocytes at 220 mOsm prior to the osmotic challenge.

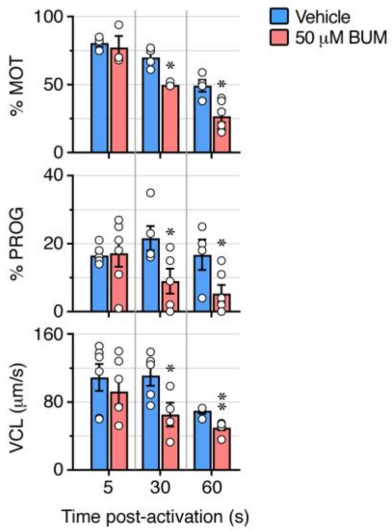

Figure S4. Inhibition of NKCC1 impairs the sperm motion kinetics. Inhibition of the percentage of motility and progressivity (% MOT and % PROG, respectively) and curvilinear velocity (VCL) at 5, 30 and 60 s post-activation induced by 50 µM of the NKCC1 blocker bumetanide (BUM). Control spermatozoa were treated with 0.5% DMSO. In all panels, the data are the mean ± SEM (*n* = 3-5 males, one ejaculated per male; white dots). Statistical differences within each time point were measured by an unpaired Student *t*-test (*, $p < 0.05$; **, $p < 0.01$; with respect to control sperm).

Figure 2C

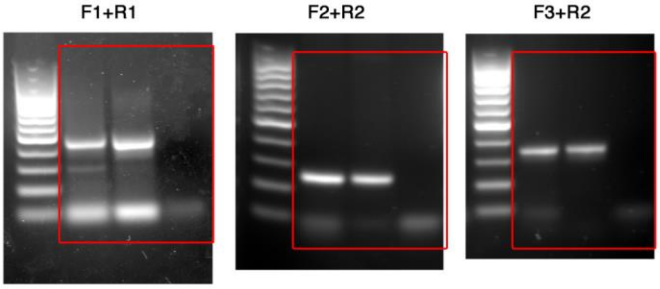

Figure 3B

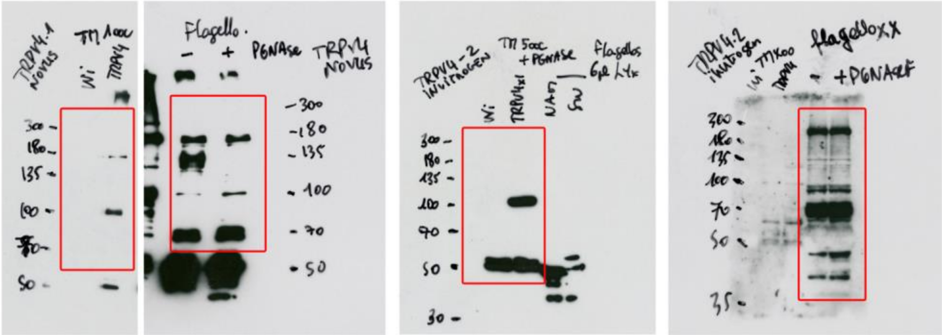

Figure 3E

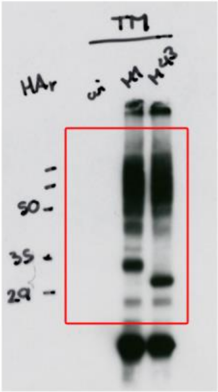

Figure 3F

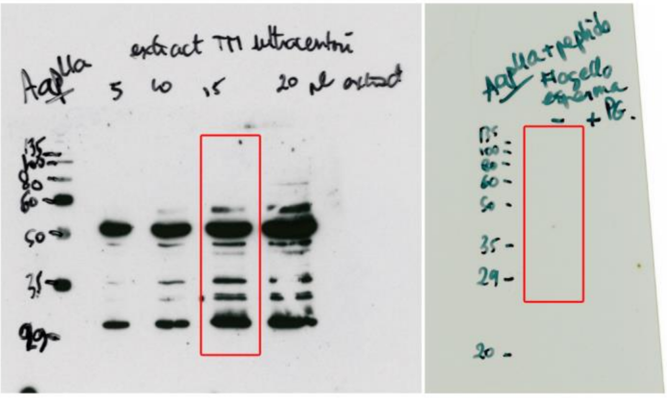

Figure 3H

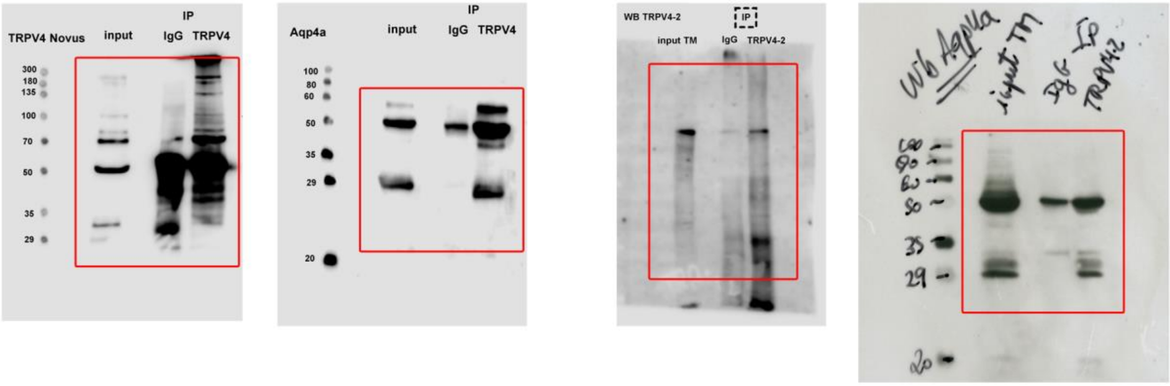

Fig. S5. Uncropped gels and immunolots from panels shown in Figs 2 and 3 as indicated.

References

Deng Z, Paknejad N, Maksaev G, Sala-Rabanal M, Nichols CG, Hite RK, Yuan P (2018) Cryo-EM and X-ray structures of TRPV4 reveal insight into ion permeation and gating mechanisms. ***Nature Structural & Molecular Biology*** 25: 252-260
