# Supplementary material for: Aqp4a and Trpv4 mediate regulatory cell volume increase for swimming maintenance of marine fish spermatozoa
Source: Cell Mol Life Sci. 2024 Jul 6;81(1):285. doi: 10.1007/s00018-024-05341-w (PMC11335209; doi:10.1007/s00018-024-05341-w)
Supplement: Supplementary file 2 — Supplementary Material 2 [file 18_2024_5341_MOESM2_ESM.pdf]

Supplementary Table S1

List of TRPV4 accession numbers used in the phylogenetic tree

GenBank accession	Animal	Species	Rank/Grade	Order	Family
XM_005253918	Human	*Homo sapiens*	Euarchontoglires	Primates	Hominidae
XR_003569294	Wombat	*Vombatus ursinus*	Metatheria	Diprotodontia	Vombatidae
XM_029048643	Platypus	*Ornithorhynchus anatinus*	Prototheria	Monotremata	Ornithorhynchida
XM_005498200	Rock pigeon	*Columba livia*	Aves	Columbiformes	Columbidae
XM_032228806	Garter snake	*Thamnophis elegans*	Lepidosauria	Squamata	Colubridae
XM_044440427	Komodo dragon	*Varanus komodoensis*	Lepidosauria	Squamata	Varanidae
XM_041566056	African clawed frog	*Xenopus laevis*	Amphibia	Anura	Pipidae
LE102108	Hokkaido salamander	*Hynobius retardatus*	Amphibia	Caudata	Hynobiidae
XM_033957279	Gaboon caecilian	*Geotrypetes seraphini*	Amphibia	Gymnophiona	Dermophiidae
XM_005999430	Coelacanth	*Latimeria chalumnae*	Actinistia	Coelacanthiformes	Coelacanthidae
XM_030415727	Gilthead seabream	*Sparus aurata*	Euacanthomorphacea	Perciformes	Sparidae
XM_040196296	3-spined stickleback	*Gasterosteus aculeatus*	Euacanthomorphacea	Gasterosteiformes	Gasterosteidae
XM_020100862	Japanese flounder	*Paralichthys olivaceus*	Euacanthomorphacea	Pleuronectiformes	Paralichthyidae
XM_040154184	Swordfish	*Xiphias gladius*	Euacanthomorphacea	Istiophoriformes	Xiphiidae
XM_036143668	Common mummichog	*Fundulus heteroclitus*	Euacanthomorphacea	Cyprinodontiformes	Fundulidae
XM_023269883	Clown anemonefish	*Amphiprion ocellaris*	Euacanthomorphacea		Pomacentridae
XM_044371238	Yellowfin tuna	*Thunnus albacares*	Euacanthomorphacea	Scombriformes	Scombridae
XM_014160771	Atlantic salmon	*Salmo salar*	Protacanthopterygii	Salmoniformes	Salmonidae
XM_010876763	Northern pike	*Esox lucius*	Protacanthopterygii	Esociformes	Esocidae
XP_005165208	Zebrafish	*Danio rerio*	Ostariophysi	Cypriniformes	Cyprinidae
XM_030791017	Milkfish	*Chanos chanos*	Ostariophysi	Gonorynchiformes	Chanidae
XM_018748947	Asian arowana	*Scleropages formosus*	Osteoglossomorpha	Osteoglossiformes	Osteoglossidae
XM_035435509	European eel	*Anguilla anguilla*	Elopomorpha	Anguilliformes	Anguillidae
XM_006640334	Spotted gar	*Lepisosteus oculatus*	Holostei	Semionotiformes	Lepisosteidae
XM_034925198	Sterlet	*Acipenser ruthenus*	Chondrostei	Acipenseriformes	Acipenseridae
XM_028825072	Reedfish	*Erpetoichthys calabaricus*	Cladistia	Polypteriformes	Polypteridae
XM_042337715	Ghost shark	*Callorhinchus* milii	Chondrichthyes	Chimaeriformes	Callorhinchidae

Supplementary Table S2

Oligonucleotide primers employed for RT-PCR detection of expression of the *trpv4* splice variants

Trpv4 variant	Ensembl accession no.	Forward (F) / Reverse (R)	Amplicon (bp)
Trpv4_v1	ENSSAUT00010061485.1	F1: CCTAAAGCCCTGCTGAACCT<br>R1: CGTGTTGTCCTTGGTGTTGT	432
		F2: TGCTTCAGCTTCATTTCCAG<br>R2: TCGCTCTCAAACAGCTCTGA	247
Trpv4_v2	ENSSAUT00010061488.1	F1: CCTAAAGCCCTGCTGAACCT<br>R1: CGTGTTGTCCTTGGTGTTGT	291
Trpv4_v10	ENSSAUT00010061519.1	F3: TTTCCCTGGAAGTGTTTTGG<br>R2: TCGCTCTCAAACAGCTCTGA	334
