# Supplementary material for: Aqp4a and Trpv4 mediate regulatory cell volume increase for swimming maintenance of marine fish spermatozoa
Source: Cell Mol Life Sci. 2024 Jul 6;81(1):285. doi: 10.1007/s00018-024-05341-w (PMC11335209; doi:10.1007/s00018-024-05341-w)
Supplement: Supplementary file 3 — Supplementary Material 3 [file 18_2024_5341_MOESM3_ESM.doc]

TRPV4 alignment

>Human_TRPV4

------------------MADSSEGPR------AGPGEVAELPGDESGTPG-GEAFP-LS

SLANLFEGEDGSLSPSPADAS----------RPA--GPGDGRPNLRMKFQGAFRKGVPNP

IDLLESTLYESSVVPGPKKAPMDSLFDYGTYRHHSSD-NKRWRKKII-EK---QPQS-P-

---KAPAPQPPPILKVFNRPILFDIVSRGSTADLDGLLPFLLTHKKRLTDEEFREPSTGK

TCLPKALLNLSNGRNDTIPVLLDIAERTGNMREFINSPFRDIYYRGQTALHIAIERRCKH

YVELLVAQGADVHAQARGRFFQPKDEGGYFYFGELPLSLAACTNQPHIVNYLTENPHKKA

DMRRQDSRGNTVLHALVAIADNTRENTKFVTKMYDLLLLKCARLFPDSNLEAVLNNDGLS

PLMMAAKTGKIGIFQHIIRREVTDEDTRHLSRKFKDWAYGPVYSSLYDLSSLDTCGEEAS

VLEILVYNSKIENRHEMLAVEPINELLRDKWRKFGAVSFYINVVSYLCAMVIFTLTAYYQ

PLEGTPPYPYRTTVDYLRLAGEVITLFTGVLFFFTNIKDLFMKKCPGVNSLFIDGSFQLL

YFIYSVLVIVSAALYLAGIEAYLAVMVFALVLGWMNALYFTRGLKLTGTYSIMIQKILFK

DLFRFLLVYLLFMIGYASALVSLLNPCANMKV-CNEDQTNCTVPTYPSCRDSETFSTFLL

DLFKLTIGMGDLEMLSSTKYPVVFIILLVTYIILTFVLLLNMLIALMGETVGQVSKESKH

IWKLQWATTILDIERSFPVFLRKAFRSGEMVTVGKSSDGTPDRRWCFRVDEVNWSHWNQN

LGIINEDPGKN--ETYQYYG-FSHTVGRLRRDRWSSVVPRVVELNKNSN-PDEVVVPL--

-DSMGNPRCDGH-QQGYPRKWRTDDAPL-------------------------

>Wombat_TRPV4

------------------MTDPEDPPR------ASPGESGEPPGDD-SGPP-GETFP-LS

SLANLFEGEDGSPSAGGEPSR----------SPP--AQGDARQNLRMKFHGAFRKGMPNP

MDLLESTIYESSVVPGPKKAPMDSLFDYGTYRHHPSDNNKRRRKKII-EK---EPQN-P-

---KAPAPDPPPILKVFNRPILFDIVSRGSTADLDGLLSFLLTQKKRLTDEEFREPSTGK

TCLPKALLNLSGGRNDTIPVLLDIAERTGNMREFINSPFRDVYYRGQTALHIAIERRCKH

YVELLVAQGADVHAQARGRFFQPKDEGGYFYFGELPLSLAACTNQPHIVNYLTENPHKKA

DMRRQDSRGNTVLHALVAIADNTRENTKFVTKMYDLLLIKCAKLFSESNLEAVVNNDGLS

PLMMAAKTGKIGIFQHIIRREVTDEDARHLSRKFKDWAYGPVYSSLYDLSSLDTCGEETS

VLEILVYNSKIENRHEMLAVEPINELLRDKWRKFGAVSFYISVVSYLCAMIIFTLTAYHQ

PLEGTPPYPYRTTVDYLRLAGEIITLSTGVIFFLSSIKDLFMKKCPGVNSLFVDGSFQLL

YFIYSVLVIVSAGLYLAGIEAYLAVMVFALVLGWMNALYFTRGLKLTGTYSIMIQKILFK

DLFRFLLVYLLFMIGYASALVSLLNPCSSNAI-CNEDRSNCTVPSYPSCRDSQTFSTFLL

DLFKLTIGMGDLEMLSSAKYPVVFIILLVTYIILTFVLLLNMLIALMGETVGQVSKESKH

IWKLQWATTILDIERSFPVFVRKAFRSGEMVTVGKSSDGTPDRRWCFRVDEVNWSHWNQN

LGIINEDPGKN--ETYQYYG-FSHTVGRLRRDRWSVVVPRVVELNKNTQ-SEDVVVPL--

-DHVGTSSAPAQ-RQSYPHHWRTEDAPL-------------------------

>Platypus_TRPV4

------------------MAEPDEPPR--------TGDTGEPLGDE-ASPP-GEAFP-LS

SLANLFEGEDGGTPVQPGAGP----------PPP--APGDTKQNLRMKLHGAFRKGVSSP

MDLLEATIYESSVVPCPKKAPMDSLFDYGTYRHHPSD-NRRRRKKLV-EK---ETPS-V-

---KAPAPHPPPILKVFNRPILFDIVSRGSTADLDGLLSFLLTHKKRLTDEEFREPSTGK

TCLPKALLNLCNGRNDTIPLLLDIAERTGNTREFINSPFRDVYYRGQTALHIAIERRCKH

YVELLVALGADVHAQARGRFFQPKDEGGYFYFGELPLSLAACTNQPHIVNYLTGNPHKKA

DLRRQDSRGNTVLHALVAIADNTRDNTKFVTKMYDLLLVQCTKLFPDSHLETVLNNDGLS

PLMMAAKTGKIGVFQHIIRREVKDEEVRHLSRKFKDWAYGPVYSSLYDLSSLDTCGEEAS

VLEILVYNSKIENRHEMLAVEPINELLRDKWRKFGAVSFYISVVSYLCAMVIFTLTAYYR

PLQGPPPYPYQTPMDYLRLAGELVTVFTGVLFFFTNIKDLFMKKCPGVNSLFIDGSFQLL

YFIYSVLVMVSAGLYLGGVEAYEAVMVFALVLGWMNALYFTRGLKLTGTYSIMIQKILFK

DLFRFLLVYLLFMIGYASALVSLLNPCPGREA-CPRGQSNCTVPTYPSCRDSETFSVFLL

DLFKLTIGMGDLEMLGSTKYPVVFVILLVTYIILTFVLLLNMLIALMGETVGQVSKQSKH

IWKLQWATTILDIERSFPVFVRKAFRSGEMVTVGKGVDGTPDRRWCFRVDEVNWSHWNQN

LGIINEDPGKN--ETYQYYG-FSHTVGRLRRDRWSTVLPRVVELNKNSH-PDDVVVPL--

-DQLGGSGTPAPGRSTYPATWRTEDAPL-------------------------

>Rock_pigeon_TRPV4

------------------MADAEDPPR------ALGGDAGEGPGDD-GSLQ-NDSFP-LS

SLANLFESEDTPSPAEAAR------------GPP--GTGDGKQNLRMKFHGAFRKGAPKP

MELLEATIYESSVVPAPKKAPMDSLFDYGTYRHHPSE-NKRWRRRVV-EK---QAPG-G-

---KGPAPNPPPVLKVFNRPILFDIVSRGSPAGLDGLLSFLLTHKKRLTDEEFREPSTGK

TCLPKALLNLSGGRNDTIPILLDIAEKTGNMREFINSPFRDVYYRGQTALHIAIERRCKH

YVELLVEKGADVHAQARGRFFQPKDEGGYFYFGELPLSLAACTNQPHIVHYLTENGHKQA

DLRRQDSRGNTVLHALVAIADNTRENTKFVTKMYDLLLVKCAKLFPDTNLEVLLNNDGLS

PLMMAAKTGKIGIFQHIIRREITDEDARHLSRKFKDWAYGPVYSSLYDLSSLDTCGEEVS

VLEILVYNSKIENRHEMLAVEPINELLRDKWRKFGAVSFYISVVSYLCAMVIFTLVAYYR

PMEGPPPYPYTTTVDYLRLAGEIITLLTGILFFFTNIKDLFMKKCPGVNSFFIDGSFQLL

YFIYSVLVIVTAGLYLGGIEAYLAVMVFALVLGWMNALYFTRGLKLTGTYSIMIQKILFK

DLFRFLLVYLLFMIGYASALVSLLNPCPSSES-CSQEQPNCSVPTYPSCRDSQTFSTFLL

DLFKLTIGMGDLEMLESAKYPGVFIILLVTYIILTFVLLLNMLIALMGETVGQVSKESKH

IWKLQWATTILDIERSFPVFLRKAFRSGEMVTVGKGTDGTPDRRWCFRVDEVNWSHWNQN

LGIISEDPGKS--DTYQYYG-FSHTVGRLRRDRWSTVVPRVVELNKSCQ-PEEVVVPL--

-GTVGTAEARER-RHGQASG-----SPL-------------------------

>Garter_snake_TRPV4

------------------MADPEDTPV------ASPAEAPEAPAEE-SSPQ-GEAFP-LS

SLANLFEGEEGSGPGEAPR------------SPP--GSGDGKQNLRMKFHGAFRKGVPNP

MELLESTIYESSVVPGPKKAPMDSLFDYGTYRHLPSD-NKRRRKRAL-EK---KRPS-A-

---KGPAPNPPPILKIFNRPILFDIVSRGSLDGLDGLLPFLLGHKKRLTDEEFRELSTGK

TCLPKALLNLNGGRNDTIPLLLDIAEKTGNMREFINAPFRDVYYRGQTALHIAIERRCKH

YVELLVEKGADVHAQARGRFFQPKDEGGYFYFGELPLSLAACTNQPHIVQYLTENAHKQA

DLRRQDSRGNTVLHALVAIADNTRENTKFVTKMYDLLLIKCAKLFPDTNLEALLNNDGLS

PLMMAAKTGKIGMFQHIIRREVKDEEARHLSRKFRDWAYGPVYSSLYDLSSLDTCGEEVS

VLEILVYNSKIENRHEMLAVEPINELLRDKWRKFGAVSFYISVVSYLCAMVIFTLVAYYR

PLEGTPPYPYTTTPDYLRLAGEIVTLFTGVLFFFTNVKDLFMKKCPGVNSFFIDGSFQLL

YFIYSVLVLVAAALYLAGIEAYLAVMVFALVLGWINALYFTRGLKLTGTYSIMIQKILFK

DLFRFLLVYVLFMIGYASALVSLLNPCPSAES-CRGERSNCTAPAYPSCRDSKTFSTFLL

DLFKLTIGMGDLEMIENAKYPGVFVILLVTYIILTFVLLLNMLIALMGETVGQVSKESKK

IWKLQWATTILDIERSFPVFVRRAFRSGEMVTVGKSLDGAPDRRWCFRVDEVNWSHWNQN

LGIINEDPGKN--DTYQYYG-FSHTVGRLRRDRWSTVVPRVVELNKNSQ-PDEVVVPL--

-DSLRPAGANAH-KPSYPHSWRKEDAQI-------------------------

>Komodo_dragon_TRPV4

------------------MADSEDSPH------PSPGEATDPPAED-ASPQ-NDAFP-LS

SLANLFEGEEGSS--EAPR------------GPP--GSGDGKQNLRTKLHGAFRKGVPNP

MDLLEATIYESAVVPGPKKAPMDSLFDYGTYRHHPSD-NRRRRKKAP-ER---KPPS-T-

---KGPAPHPPPVLKVFNRPILFDIVSRGSTAGLDGLLPFLLTHKKRLTDEEFREPSTGK

TCLPKALLNLSSGRNDTIPVLLDIAEKTGSMREFINSPFRDVYYRGQTALHIAIERRCKH

YVELLVEKGADVHAQARGRFFQPKDEGGYFYFGELPLSLAACTNQPHIVHYLTENAHKQA

DLRRQDSRGNTALHALVAIADNTRENTKFVTKMFDLLLIKGAKLFPDTSLEALLNNDGLS

PLMMAAKTGKIGIFQHIIRREIKDEDARHLSRKFKDWAYGPVYSSLYDLSSLDTCGEEVS

VLEILVYNSKIENRHEMLAVEPINELLRDKWRKFGAASFYISVVSYLCAMVIFTLVAYYR

PLEGIPPYPYTSTTDYLRLAGEVVTLSTGVLFFFTNIKDLFLKKCPGVNSFFIDGSFQLL

YFIYSVLVLVAAALYLAGIEAYLAVMVFALVLGWMNALYFTRGLKLTGTYSIMIQKILFK

DLFRFLLVYVLFMIGYASALVSLLNPCPSLEA-CGEDRANCTAPAYPSCRDSRTFSAFLL

DLFKLTIGMGDLEMIESAKYPGVFVILLVTYIILTFVLLLNMLIALMGETVGQVSKESKQ

IWKLQWATTILDIERAFPVFVRKAFRSGEMVTVGKALDGTPDRRWCFRVDEVNWSHWNQN

LGIINEDPGKS--DTYQDYG-FSHTVGRLRRDRWSTVVPRVVELNKNAH-PEEVVVAL--

-ESVRGPQAKE--KPSYPQGWRRDDSHI-------------------------

>African_clawed_frog_TRPV4

------------------MADPSYLLK-----PNASVDIDDYQGDD-GSTH-NDSFP-LS

SLANLFENEEGSAPNEGVR------------SPQ--VP-DNKQNLRIRFQGPFRKGISNP

MDLLESTIYESS---APKKAPMDSLFGYETYHHHPTE-NRRKRKKIHLEK---ENLN-S-

---QASSPDPPPVIKMFNRHILFDIVSRGCTAELEGFLPFLLAQKKRLTDEEFREASTGK

TCLTKALMNLNGGKNDTIPMLIDIAEKTGNLREFINSPFRDVYYRGQTALHIAIERRCKH

YVELLVEKGADVHAQARGRFFQPKDEGGYFYFGELPLSLAACTNQPDIVHYLTENVHKKA

DIRRQDSRGNTVLHALVAIADNTRENTKFLTKIYDLLVIKCVKLYPDCSLEAIFNNDSMS

PLMMAAKLGKIGIFQHIIRLEIKDEEARHLSRKFKDWAYGPVYSSLYDLSMLDTCGEEVS

VLEILVYNSKVENRHEMLAVEPINELLRDKWQKFGAVSFYISVISYLIAMIIFTLIAYYR

PMDGTPPYPYRTTMDYMRLAGEIITLLTGVVFFITNIKDLFMKKCPGVNSLFIDGSFQLL

YFIYSVLVIITAVLYLVGIESYLAVMVFALVLGWMNALYFTRGLKLTGTYSIMLQKILFK

DLFRFLLVYLLFMIGYASALVSLLNPCTNQEI-CLEANTNCTVPEYPSCRDSSTFSKFLL

DLFKLTIGMGDLEMINSAKYPAVFIILLVTYIILTFVLLLNMLIALMGETVGQVSKESKQ

IWKLQWATTILDIERSFPVCMRKAFRSGEMVTVGKNLDGTPDRRWCFRVDEVNWSHWNQN

LGIINEDPGRN--DNYQYYG-FSQTVGRLRRDRWSVVVPRVVELNKAPQHSDDVVVPL--

-GHIPQVQTYYQ-RQENAQNWKKDETHI-------------------------

>Hokkaido_salamander_TRPV4

------------------MTDSDDPVR-----LNSSMDVNDSQNDD-GNAT-NESFP-LS

SLANLFENEDGTPAIEPTR------------TPQ--ASGDNKQNLRMKFQGAFKKGMPNP

MDLLESTIYESSVVPGPKKAPMDSLFDYETYHHHPTE-NKRRRKKINADK---QNQS-P-

---KSQAPDPPPVLKVFNRHILFDVVSRGSTSDLDGLLSFLLTQKKRLTDEDFREASTGK

TCLPKALMNLCGGKNDTIPMLMDIAEKTGNLREFINAPFRDVYYRGQTALHIAIERRCKH

YVELLVEKGADVHAQARGRFFQPRDEGGYFYFGELPLSLAACTSQPDIVRYLTENAHKKA

DLRRQDSRGNTVLHALVAIADNTKENTKFVTKMYDLLVIKCVKLFPDCSLESFFNNDGLS

PLMMAAKLGKIGVFQHIIRREIKDEDARHLSRKFKDWAYGPVYSSLYDLSSLDTCGEEVS

VLEILVYNSKIENRHEMLAVEPINELLRDKWRKFGAVSFYISVVSYLIAMIIFTLTAYYR

PLDGIPPYPYTTTIDYLRLAGEIITLFMGILFFFTNIKDLFMKKCPGVNSLFIDGSFQLL

YFIYSVLVIVTAALYLAGIEAYLAVMVFALVLGWMNALYFTRGLKLTGTYSIMIQKILFK

DLFRFLLVYLLFMIGYASALVSLLNPCPGTI--CLENATNCTTPEYPSCRDSSTFSKFLL

DLFKLTIGMGDLEMINSAKYPAVFIILLVTYIILTFVLLLNMLIALMGETVGQVSKESKQ

IWKLQWATTILDIERSFPVFVRKAFRSGEMVTVGKSLDGAPDRRWCFRVDEVNWSHWNQN

LGIINEDPGKN--ETYQYYG-ISQTVGRLRRDRWSSVVPRVVELNKNPH-SDEVVVSL--

-DQMRPSNIPYE-REGCTQNWKRDETHI-------------------------

>Gaboon_caecilian_TRPV4

------------------MADSEDPPK-----ANTSVD-GEAEKEE-GSSQ-NDSFP-LS

SLANLFENEDGTPSADPSR------------TPQ--AAGDNKQNLRMKFQGAFKKGMPNP

MDLLESTIYESSVAPAPKKAPMDSLFDYGTYYHHPTE-NKRRRRKINADK---QTQS-S-

---KAPAPDPPPVLKVFNRHILFDIVSRGSTADLDGLLSFLLTQKKRLTDEEFREPSTGK

TCLPKALLNLNGGRNDTIPMLVNIAEKTGNLREFINSPFRDVYYRGQTALHIAIERRCKH

YVELLVEKGADVHAQARGRFFQPKDEGGYFYFGELPLSLAACTNQPDIVHYLTENVHKKA

DLRRQDSRGSTVLHALIAIADNTKENTKFVTKMYDLLVIKCCKLYPDCNLEAFFNNDGLS

PLMMAAKLGKIGVFQHIIRREIKDEDARHLSRKFRDWAYGPVYSSLYDLSSLDTCGEEVS

VLEILVYNSKIENRHEMLAVEPINELLRDKWRKFGAVSFYISVVSYLIAMIIFTLIAYYR

PLEGPPPYPYNSMRDYIRLAGEIITLCTGILFFFSNIKDLFMKKCPGVNSLFIDGSFQLL

YFIYSVLVIITAALYLAGIEAYLAVMVFALVLGWMNALYFTRGLKLTGTYSIMLQKILFK

DLFRFLLVYVLFMIGYASALVSLLKPCAGEEF-C-HNVTNCTLPEYPSCRDSSTFSKFLL

DLFKLTIGMGDLEMINSAKYPAVFIILLVTYIILTFVLLLNMLIALMGETVGQVSKESKQ

IWKLQWATTILDIERFFPVFVRKAFRSGEMVTVGKNLDGTPDRRWCFRVDEVNWSHWNQN

LGIINEDPGKN--DTYQYYG-LSQTVGRLRRDRWSSVVPRVVELNKQPH-LDDVVVPL--

-DYMGAPNTH-------DQNWKRDDTHI-------------------------

>Coelacanth_Trpv4

------------------MGDVEEQNK-----LNSSGDANDTRTED-GSQQ-NDAFP-LS

SLANLFENEEGATATDTAK------------APQ--TAGDGKQNLRMKFHGAFKKGMPNP

MDLLESTIYESPVVPAPKKAPMDSLFDYGTYHQYPTE-NKRRRKRIIHDK---QSPN-Q-

---KTQAPNPPPILKVFNRPILFDIVSRGTAAELDGLLAFLVTHKKRLADEEFREPSTGK

TCLPKALLNLTNGKNDTIPILVEIAEKTGNLREFINSPFRDVYYRGQTALHIAIERRCKQ

YVELLVEKGADVHAQARGRFFQPRDEGGYFYFGELPLSLAACTNQPDIVHYLIENAHKKA

DLRRQDSRGNTVLHALVAIADNTRENTKFVTKMYDMLLIKCAKLFPECNLEAILNNDGMS

PLMMAAKLGKIGIFKYIIRREVMDENARHLSRKFKDWAYGPVYSSLYDLSSLDTCGMEVS

VLEILVYNSRIENRHEMLAVEPINELLRDKWRKFGAVSFYISVVSYLVAMVIFTLIAYYR

PVEGRPPYPYKTTIDYLRLAGEVITLLTGIFFFFTNIKDLFLKKCPGVNSLLIDGSFQLL

YFIYSVLVLVTAALYLTGIEAYVAVMVFALVLGWMNTLYFTRGLKLTGTYSIMLQKILFK

DLFRFLLVYVLFMIGYASALVSLLNPCLTTET-CTANNASCTAPEYPYCRDSNTFGKFIM

DLFKLTIGMGDLEMVNSAKYPGVFIILLVTYIILTFVLLLNMLIALMGETVGQVSKESKQ

IWKLQWATTILDIERSFPVFLRRVFRSGEMVTVGKNLDGTPDRRWCFRVDEVNWSHWNQN

LGIINEDPGKN--ETYQYYG-FSHTMGRLRRDRWSTVVPRVVELNKTPR-ADEVVVSL--

-DPMGMSNVHDT-KRGDPHNWKKDETQI-------------------------

>Gilthead_seabream_Trpv4

MN-EGRS--ALLRRCNLALSKANALGSV---PAGAAVSVDAGDGGA-AQPESDAAFV-LS

EFSELFESEDASPSTQDTSQGSALELVQP-GQPG--QPADGRQNLRMKFHGAFKKGISNP

MDLLESTIYESNVVQGPKKAPMDSLFDYGTYRN-TSN-QKRRRKKLPRGKT--ETSC-D-

---DAQSSDPPKVMKIFNRSLLFDCVSRADIEALEGLLEYLQSHDKRLTDEDFREPSTGK

TCLPKALLNLYGGQNVTIPLLVDIAEKTGNLREFINTPFRDVYYRGQTALHIAIERRCKQ

YVELLVEQGADVHAQARGRFFQPRDEGGYFYFGELPLSLAACTNQPDIVHYLTENPHKKA

DLRRQDSRGNTVLHALVHIADNTKDNTRFLTKMYDLLLIKSAKLYPDCNLERVLNNDCMS

PLMMAAKLGKIGVFQHIIRREIKDEEARHLSRKFKDWAYGPVYSSLYDLSSLDTCGEEPS

VLEILVYNSRNENRHEMLAVEPINELLRAKWQKFGAVTFYISVVSYLITMIIFTLVAYYH

PTQGKPPYPYTTSSDYLRMAGEIVTLASGIFFFLTNIKDLFLKKCPGVKSLFIDGSFQLL

YFIYSVLIVVTAALYLSGIKAYVSVMVFALVLGWMNTLYFTRGLKLTGTYSIMIQKILFK

DLFRFLLVYVLFMIGYASALVSLLTVCPPPGTEC---DGDC--PTYPNCRDPDTFSTFLL

DLFKLTIGMGELDMIHSAQYPAVFLILLVTYIILTFVLLLNMLIALMGETVGQVSKESKK

IWKLQWATTILDIERSFPVCLRRSFRAGEMVTVGKNCDGTPDRRWCFRVDEVNWCHWNQN

LAIINEDPGKN--ETCQVNG-LQQSVRALRRDRWSTVVPRAVELSKSPR-PHDLVLEM--

-EPLTPRH---------------------------------------------

>3_spined_stickleback_Trpv4

MN-EGRS--ALFRRCHLALSKADTVGSV---PGRNAASVDSGDGAA-PQPDGDAALG-LS

ELSHLFENDDGSPSTQDTSGGSAPELV----QPG--QPAEGRQNLRMKFQGAFRKGISTH

MDLLESTIYESNVVQGPKKAPMDSLFDYGTCRN-TSN-QKRRRKKLPRGKT--EASCDD-

---DGQSSDPPKVMKVFNRSLLFDAVSRADPEALEGLLEYLQSHEKRLTDEEFKELSTGK

TCLPKALLNLYGGQNVTIPLLVDVAEKTGNLREFINTPFRDVYYRGQTALHIAIERRCKQ

YVELMVEHGADVHAQARGRFFQPRDEGGYFYFGELPLSLAACTNQPNIVHYLTENPHKKA

DLRRQDSRGNTVLHALVHIADNTKDNTRFLTKMYDLLLVKSAKLYPDCNLETVLNNDGMS

PLMMAAKLGKIGVFQHIIRREIKDEEVRQLSRKFKDWAYGPVYSSLYDLSSLDTCGEEPS

VLEILVYNSRNENRHEMLAVEPINELLRAKWQKFAAVTFYISVVSYLITMIIFTLVAYYH

PTQGTPPYPYTTSSDYLRMAGEILTLASGIFFFLTNIKDLFLKKCPGVKSLFMDGSFQLL

YFIYSVLIVVTAALYLSGIKAYVSVMVFALVLGWMNTLYFTRGLKLTGTYSIMIQKILFK

DLFRFLLVYVLFMIGYASALVSLLTMCPPPGTEC---EGGC--PTYPKCRDPDTFSAFLL

DLFKLTIGMGELDMIHGAQYPAVFLILLVTYIILTFVLLLNMLIALMGETVGQVSKESKK

IWKLQWATTILDIERSFPVCLRKSFRAGEMVTVGKSWDGTPDRRWCFRVDEVNWCHWNQN

LAIINEDPGKN--EVCQANG-LQQGVRALRRDRWSTVVPRAVELSKGPR-PRDLSIEM--

-EPLTPRH---------------------------------------------

>Japanese_flounder_Trpv4

MN-EGRS--ALFRRCHLALSKADTLTSA---PAKADLSVDAGDGEA-AQPEADAAFP-LS

DLSHLFESEDAPPSTQDTSQDSALELV----QPG--QPVDSRQNLRMKFQGAFKKGISDP

MDLLESTIYESNVVQGPKKAPMDSLFDYGTYSD-MSN-QKRRRKKLPRGKT--EMSC-D-

---DGQ-TDPPKVMKTFNRSVLFDCVSRGDPGALDGLLEYLQSHGKRLTDEEFREPSTGK

TCLPKALLNLYGGQNDTIPLLVDIAEKTGNLREFINSPFRDLYYRGQTALHIAIERRCKQ

YVELLVENGADVYAQARGRFFQPRDEGGYFYFGELPLSLAACTNQPDIVHYLTENPHKKA

DLRRQDSRGNTVLHALVHIGDNTKDNTRFLTKMYDLLLIKSAKLYPECSLETVLNSDCMS

PLMMAAKLGKIGVFQHIIRREIKDEEVRHLSRKFKDWAYGPVYSSLYDLSSLDTCGEEPS

VLEILVYNSRNENRHEMLAVEPINELLRAKWQKFGAVTFYISVVSYLITMIIFTLVAYYH

PTQGEPPYPYKTSSDYTRMAGEVVTLASGIFFFLTNIKDVFLKKCPGVKSLFIDGSFQLL

YFIYSVLIVVTAALYLSGIKAYVSVMVFALVLGWMNTLYFTRGLKLTGTYSIMIQKILFK

DLFRFLLVYVLFMIGYASALVSLLTVCPPPGTKC---DDGC--PTYPKCRDPDTFSTFLL

DLFKLTIGMGELDMIHSAQYPAVFLILLVTYIILTFVLLLNMLIALMGETVGQVSKESKK

IWKLQWATTILDIERSFPVCLRKSFRAGEMVTVGKNWDGTPDRRWCFRVDEVRWCHWNQN

MAIISEDPG-------QVNV-QQQGVGVLRRDRWSTVVPRVVELNKG----REAVVEM--

-EPLTRRR---------------------------------------------

>Swordfish_Trpv4

MN-EGRS--ALFRRGHLALSKADTLSSV---SARGAPSVDRGDGGA-AQPESDAAFP-LS

EFSHLFESMDASPSDKDASQDSAPELV----QPG--QPADSRQNLRMKFQGAFKKGISNP

MDLLESTIYESSVVPGPKKAPMDSLFDYGTYGN-MSN-QKRRRKKLPRGKT--ETSS-D-

---DGQSSDPPKVMKTFNRSLLFDCVSRGDPGALEGLLEYLQSHGKRLTDEEFREPSTGK

TCLPKALLNLYGGQNATIPLLVDIAEKTGNLREFINSPFRDLYYRGQTALHIAIERRCKQ

YVELLVEKGADVHAQARGRFFQPKDEGGYFYFGELPLSLAACTNQPDIVHYLTEYPQKKA

DLRRQDSRGNTVLHALVHIADNTKDNTRFLTKMYDLLLIKSAKLYPECSLETVLNNDGMS

PLMMAAKLGKIGVFQHIIRREIKDEEVRHLSRKFKDWVYGPVYSSLYDLSSLDTCGEEPS

VLEILVYNSRNENRHEMLAVEPINELLRAKWQKFAAVTFYISVVSYLITMIIFTLVAYYH

PTQGTPPYPYTTSSDYLRMAGEIVTLASGIFFFLTNIKDVFLKKCPGVKSLFIDGSFQLL

YFIYSVLIVVTAALYLSGIKAYVSVMVFALVLGWMNTLYFTRGLKLTGTYSIMIQKILFK

DLFRFLLVYVLFMIGYASALVSLLTVCPRPGTEC---HGDC--PTYPKCRDPDTFSTFLL

DLFKLTIGMGELDMIHSAQYPAVFLILLVTYIILTFVLLLNMLIALMGETVGEVSKESKK

IWKLQWATTILDIERSFPVCLRKSFRAGEMVTVGKNWDGTPDRRWCFRVDEVHWCHWNQN

LAIINEDPGKS--ETSQANV-LQQSVGVLRRDRWSTVVPRVVELSKG----REAVIEM--

-EPLTRRR---------------------------------------------

>Common_mummichog_Trpv4

MN-EGRS--ALLRRCHLVLSKTDTIGAP---PVRSAPSEDAGDGVA-AQAENDAAFP-LS

ELSHLFESDDGSQSAQDTSLDAALEQAQPSAQPG--HPSDSRQNLRMKFQGAFKKGISNP

MDLLETTIYESN-VPGPKKAPMDSLFDYGTYGS-SSN-QKKRRKKLPRGKT--ETSC-D-

---DSQSSDPPKVLKIFTRFVLFDCVSRGDPQALEGLLEYLQSQEKRLTDEEFRELSTGK

TCLPKALLNLYGGRNDTIPVLVDIAEKTGNLREFINTPFRDVFYRGQTALHIAIERRCKQ

YVELLMEKGADVHAQARGRFFQPKDEGGYFYFGELPLSLAACTNQPDIVHYLTENPHKKA

DLRRQDSRGNTVLHALVHIADNTKDNTRFLTKMYDLLLIKCAKLFPDCNLETILNNDGMS

PLMMAAKLGKIGVFQHIIRREIKDEEVRHLSRKFKDWAYGPVYSSLYDLSSLDTCGEEPS

VLEILVYNSRNENRHEMLAVEPINELLRAKWQKFAAATFYISVVSYLISMIIFTLVAYYH

PTQETPPYPYNTSSDYLRMTGEIVTLASAIFFFLTNVKNVFLKKCPEVKSLVFDGSFQLL

FLIYAVLIIVTAALYLSGIKAYVSVMVFALVLGWMNTLYFTRGLKLTGTYSIMIQKILFK

DLFRFLLVYVLFMIGYASALVSLLPVCP---TEC---VGEC--PTYPSCRKPDTFSIFLL

DLFKLTIGMGDLDMIHSALYPGVFLILLVTYIILTFVLLLNMLIALMGETVGQVSKESKK

IWKLQWATTILDIERSFPVCLRKSFRAGEMVTVGKNVDGSPDRRWCFRVDEVNWCHWNQN

LAIISEDPGKT--ETSQTNG-LRQSARGLRRDRWSTVVPRAVELSRSPR-SNDLVVEM--

-EPLTPRH---------------------------------------------

>Clown_anemonefish_Trpv4

MN-ESRS--ALLRRCHLVLSKADTLGSV---PARAAPSEDSEDGVA-AQPESDAAFP-LS

DLSHLFESEDGSPSTQDTSQDSVLELV----QPG--QPVDSRQNLRMKFQGAFKKGISNP

MDLLESTIYESSVVPGPKKAPMDSLFDYGTYRN-TSN-QKRRRKKLPRGKT--EASC-D-

---DSQSSDPPKVMKIFNRPLLFECVSRGDPEALEGLLEYLQSNEKRLTDEEFREPSTGK

TCLPKALLNLYGGQNSTIPLLVDIAEKTGNLREFINTPFRDVYYRGQTALHIAIERRSKH

YVELLVEQGADVHAQARGRFFQPRDEGGYFYFGELPLSLAACTNQPDIVHYLTENPHKKA

DLRRQDSRGNTVLHALVHIADNTKDNTRFLTKMYDLLLIKTAKLYPDCSLETVLNNDGMS

PLMMAAKLGKIGVFQHIIRREIKDEEVRHLSRKFKDWAYGPVYSSLYDLSSLDTCGEEPS

VLEILVYNSRNENRHEMLAVEPINELLRAKWQKFAAVTFYISVVSYLITMIIFTLVAYYH

PTQGTPPYPYTTSSDYLRMAGEIVTLASGIFFFLTNIKDVFLKKCPGVKSLFIDGSFQLL

YFIYSVLIVVTAALYLSGIKAYVSVMVFALVLGWMNTLYFTRGLKLTGTYSIMIQKILFK

DLFRFLLVYVLFMIGYASALVSLLTVCPPPGTQC---DGEC--PTYPNCRDPDTFSTFLL

DLFKLTIGMGELDMIHSAQYPAVFLVLLVTYIILTFVLLLNMLIALMGETVGQVSKESKK

IWKLQWATTILDIERSFPVCLRKSFRAGEMVTVGKSWDGTPDRRWCFRVDEVNWCHWNQN

LAIINEDPGKS--ETNQVNG-LQQSVRALRRDRWSTVVPRVVELSKGSR-PHDLVIEM--

-EPLTSRH---------------------------------------------

>Yellowfin_tuna_Trpv4

MN-EGRSA-ALLRRCQLALSKADTLGSS---SSRAAVSVDAGDGAA-AQPESDGVFP-LS

DLSDLFESEDGSPPTQDTSQ----QLV----PPG--QPADSRQNLRMKFQGAFKKGISNP

MDLLESTIYESNVVPGPKKAPMDSLFDYGTCRN-TSN-QKRRRKKLPRGKT--EMST-D-

---DGPSPDPPKVMKVFNRSLLFECVSRGDPEALEGLLEYLQSHEKRLTDEEFRELSTGK

TCLPKALLNLYSHQNDTIPLLVDIAEKTGNLREFINTPFRDVYYRGQTALHIAIERRCKQ

YVELLVEMGADVHAQARGRFFQPRDEGGYFYFGELPLSLAACTNQPDIVHYLTENPHKKA

DLRRQDSRGNTVLHALVHIADNTKDNTRFLTKMYDLLLIKSAKLYPDCSLEKVFNNDGMS

PLMMAAKLGKIGVFQHIIRREIKDEEVRHLSRKFKDWAYGPVYSSLYDLSSLDTCGEEPS

VLEILVYNSRNENRHEMLAVEPINELLRAKWQKFAAVTFYISVVSYLITMIIFTLVAYYH

PTQGTPPYPYTTSTDYLRMSGEIITLASGIFFLLTNIKDLFLKKCPGVKSLFIDGSFQLL

YFIYSVLIIVTAALYLSGIKAYVSVMVFALVLGWMNTLYFTRGLKLTGTYSIMIQKILYK

DLFRFLLVYVLFMIGYASALVSLLTVCPPPGTEC---DGGC--PTYPKCRDPDTFSTFLL

DLFKLTIGMGELDMIHSAQYPAVFLILLVTYIILTFVLLLNMLIALMGETVGEVSKESKK

IWKLQWATTILDIERSFPVCLRKSFRAGEMVTVGKNSDGTPDRRWCFRVDEVNWCHWNQN

LAIINEDPGKN--ETCQANG-LQQSVRGLRRDRWSTVVPRVVELSKGPR-PRDLVVEM--

-EPLTARH---------------------------------------------

>Atlantic_salm_Trpv4

------------------MTETDTLHSDANKAALSSGSGEGGGSGE-GQPDADGTCPDLS

ALADLFESEEGSQSPQDPAPDVD--------RPGQLQPGDSRQNLRMKFHGAFKKGISNP

MDLLESTIYESPVAPGPKKAPMDSLFDYGTYRH-TNN-KKPRRKKLPRGKT--ETSC-NE

S-LDPPGLDPPKVLKVFNRMLLFDGVSRADPEALSGLLEYLQGHEKRLTDEEFKEPSTGK

TCLPKALLNLYSGQNDTIPMLMDIAEQTVNLHEFINTPFRDVYYRGQTALHIAIERRCKQ

YVELLVEKGADVHAQARGRFFQPRDEGGYFYFGELPLSLAACTNQPNMVHYLTENAHKKA

DLRRQDSRGNTVLHALVHIADNTRDNTRFLTKMYDLLLTKCAKLYPECSLEDILNNDGMS

PLMMAAKLGKIGVFQHIIRREIKDEEARHLSRKFKDWAYGPVYSSLYDLSSLDTCGEEVS

VLEILVYNSRIENRHEMLAVEPINELLRVKWQKFAAVTFYISVVSYLVTMIIFTLVAYYR

PSQGMPPYPYTTSTDYLRLGGEVITLGSGVFFFLTNIKDLFLKKCPGVNSLFVDGSFQLL

YFIYSVLVIVTAALYLSGIEAYVSVMVFALVLGWMNTLYFTRGLKLTGTYSIMIQKILFK

DLFRFLLVYVLFMIGYSSALVSLLAVCPGPDEVCP-EEGGC--PTYPQCRDTDTFSNFLL

DLFKLTIGMGDLDMVSSAQYPAVFLILLVTYIILTFVLLLNMLIALMGETVSQVSKESKK

IWKLQWATTILDIERSFPVCLRKSFRSGEMVTVGKNWDGTPDRRWCFRVDEVNWCHWNQN

LAIINEDPGKNITETQQCSGTVHQTVRGLRRDRWSTVVPRVVEQNKGPR-PRDLVLEM--

-EPLTPRHRP---CAEG------------------------------------

>Northern_pike_Trpv4

MTFQGRSTSTQLRRYLVA--RTDTPSSAAPKAAPFSGSGEVDADEE-GQPDANGTYNELS

ALADLFESDEGSLSPPDPAKDPALDNI----RPGQLPPGDGRQNLRMKFHGAFKKGISNP

MDRLESTIYELPVAPGPKKAPMDSLFDYGSYRD-TSH-KKPRRKKLPRGKT--EVSC-EE

DVVDPPGSDPPKVVKVFNRTLLFDGVSRADPEALSGLLDYLQGHNKRLTDEEFKEPSTGK

TCLPKALLNLYGGKNDTISLLTDIAEQTSNLHEFINTPFRDVYYRGQTALHIAIERRCKH

YVKLLVEKGADVHAQARGRFFQPREEGGYFYFGELPLSLAACTNQPDMVHYLTENPHKKA

DLRRQDSRGNTVLHALVHIADNTKDNTRFVTKMYDLLLTKCVKLYPECNLEEVLNNDGMS

PLMMAAKLGKIGVFQHIIRREIKDEEARHLSRKFKDWAYGPVYSSLYDLSSLDTCGEEAS

VLEILVYNSRIENRHEMLAVEPINELLRAKWQKFAAVTFYISVVSYIVTMVIFTLVAYYR

PSQGTPPYLYSTSTDYLRLAGEVVTLCSGVFFFITNIKDLFLKKCPGVKSLFVDGSFQLL

YFIYSVLVLVTAGLYLSGVEAYVSVMVFALVLGWMNTLYFTRGLKLTGTYSIMIQKILFK

DLFRFLLVYVLFMIGYASALVSLLTVCPGPEEVCP--ASGC--LNYPHCRDTNTFSKFLL

DLFKLTIGMGDLDMASSAQYPVVFLILLVTYIILTFVLLLNMLIALMGETVGQVSKESKK

IWKLQWATTILDIERSFPVCLRKSFRAGEMVTLGKNWDGTPDRRWCFRVDEVNWCHWNQN

LAIINEDPGKNVTEALQSSGSMRQTVRGMRRDRWSTVVPRVVEQNKGTQ-LRDHVLEM--

-ESLTSRH---------------------------------------------

>Zebrafish_Trpv4

MTDQGFSASTLLKRYRLAMTESLSVSS-----PPDNSAQDSSEAAD-----GDPNFP-MS

SMAALLENDDVSQPTHELP------------RPG--QQNDQKQNMRIRFPGPFKKGVPNP

MDLLESDYTEY-----PKQAPMDSMFDYGTCRQINNN-KKGRRKKLPRGKA--EIGMSC-

---DEGSPEPP-VLKVFNRWMLFEAVSRADPRALDGLLQYLQSHEKRLTDEEFKELSTGK

TCLPKALLNLHNGQNDTIPILVDIAEQTGNLREFINTPFRDVYYRGQMALHIAIERRCKQ

YVELLVEKGADVHAQARGRFFQPRDEGGYFYFGELPLSLAACTNQPDMVHYLTENGHKKA

DLRRQDSRGNTVLHALVHIADNTRDNTRFVTKMFDLLLIKCAKLYPDCNLENILNNDGMS

PLMMAAKLGKIGVFQHIIRREIKDEEARHLSRKFKDWAYGPVYSNLYDLSSLDTCGEEVS

VLEILVYNSKIENRHEMLAVEPINELLRAKWQKFAAVTFYISVFSYLVTMIIFTLVAYYR

PSVGKPPYAYDTTEDKVRLAGEIITVGSGLFFFVTNIKDLFLKKCPGVNSIFVDGSFQLL

YFIYSVLVLVSAALYLSGIEAYVSVMVFALTLGWMNTLYFTRGLKLTGTYSIMIQKILIK

DLFRFLLVYVLFMIGYASALVSLLTICPDKDT-C---KENC--PTYPECRDTNTFSEFLL

DLFKLTIGIGDLDMLKGAQYPAVFLILLVTYIILTFVLLLNMLIALMGETVGQVSKESKK

IWKLQWATTILDIERSFPVCLRRSFRVGEMVTVGKGLDGKPDKRWCFRVDEVKWSHWNQN

LGIINEDPGQK--DLSEH----TQGGRGLRRDRWSTVVPRVVELNRGSR---DHTVEM--

-EPLTGRHRL---KSES------------------------------------

>XM_030791017_Milkfish_Trpv4

MN-EARSVSTLMRRYRLAMTEKDASFSE-NSTAAGSGTQDSAEGKD-GSTDGDPSFP-LS

SLAELLESEEGGPPLTQDNQA----------RPGPGQPTESRQNLRIKFQGAFKKGISNP

MDLLESTIYESPVVPGPKKAPMDSLFDYGTCRQ-ANN-QKRRRKKLPRGKTEMEVSC-D-

---EGMNPDPPKVLKVFNRTLLFDAVSRADPGALEGLLQYLQANEKRLTDEEFKEPSTGK

TCLPKALLNLYNGENDTIALLVDIAEKTGNLREFINTPFRDVYYRGQTALHIAIERRCKQ

YVELLVDKGADVHAQARGRFFQPRDEGGYFYFGELPLSLAACTNQPDMVHYLTENAHKKA

DLRRQDSRGNTVLHALVHIADNTRDNTRFITKMYDLLLIKSAKLYPECNLETVLNNDGMS

PLMMAAKLGKIGVFQHIIRREIKDEEARHLSRKFKDWAYGPVYSSLYDLSSLDTCGEDVS

VLEILVYNSRIENRHEMLAVEPINELLRAKWQRFGAVTFYISVVSYLVTMIIFTLVAYYR

PSEGTPPYPYTTNSDRLRLVGEVITLASGIFFFLTNIKDLFLKKCPGVNSLFIDGSFQLL

YFIYSVLVLVTAALYLSGIKAYVSVMVFALVLGWTNALYFTRGLKLTGTYSIMIQKILFK

DLFRFLLVYVLFMIGYASALVTLLAACPPPGSDC---NGTC--PTYPECRDNDTLSKFLL

DLFKLTIGMGELDMIDNAQYPVVFLILLVTYIILTFVLLLNMLIALMGETVGQVSKESKK

IWKLQWATTILDFERSFPVCLRKSFRVGEMVTVGKNWDGTPDKRWCFRVDEVKWSHWNQN

LGIINEDPGQK--EANQYD--LPQSGRGLRRDRWSTVVPRVVELSRGPR-QRDHVVEM--

-EPLTPRHRS---GS--------------------------------------

>Asian_arowana_Trpv4

------------------MTEPDPH----------TVPAGSMDSRD-GQVENDSSFP-LS

SLANLFEGEEASLTPEV--------------------P-QNKQNLRMKFQGAFKKGISNP

MDRLENTMYEANVVAGPKKAPMDTLYDYGTYHQ-PNN-QKWRRKKLPLGKSETEVSI-D-

---KGPAPEPPRVLKVFSRSILFDAVSRADPGALEGLLEYLRVQEKSLTDEEFKEPSTGK

TCLPKALLNLYGGQNDTIPLLVDIAEQTGNLCAFINTPFRDVYYRGQTALHIAIERRSKQ

YVKLLVEKGADVHAQARGRFFQPKDEGGYFYFGELPLSLAACTNQPEMVHYLTENAHKKA

DLRRQDSRGNTVLHALVHIADNTRDNTRFLTKMYDLLLVKSARLYPDYNLETVLNNDGMS

PLMMAAKLGKIGVFQHIIGREIKDEEARHLSRKFKDWAYGPVYSSLYDLSSLDTCGEEVS

VLEILVYNSRIENRHEMLAVEPINELLREKWRKFGAISFYISVVSYLVTMIIFTLVAYYR

PSEGTPPYSYTTSSDYLRLAGEFVILLSGFFFFFSNVKDLFLKKCPGVNSLFVDGSFQLL

YFIYSVLVLVTAILYLSGVKAYITVMVFALVLGWMNTLYFTRGLKLTGTYSIMIQKILFK

DLFRFLLVYVLFMIGYASALVSLLTACPTLDSQCP--PDGC--PVYPKCRDNTTFSTFLL

DLFKLTIGMGELDMIDNAQYPAVFLTLLVTYIILTFVLLLNMLIALMGETVGQVSKESKK

IWKLQWATTILGIEGSFPVCLRKSFRVGEMVTVGKNHDGTPDRRWCFRVDEVNWSHWNQN

LGIINEDPGKN--EAL-VNG-IQQTIRGLKRDRWSTVVPRVVELHKSPP-QQDMVLEM--

-EPLNTGRRN---RPGSDSHS--------------------------------

>European_eel_Trpv4

------------------MTEPDVPPT-----------GVAGGGGE-VPAENDASFP-LS

SLANLFESEETPPSAAAAAPQ----------PPP--RPGENRQNLRMKFQGAFKKGPSNS

TDPLESSSYESPVVIGPKKAPMDSLFDYGTYRQ-PAN-QKRKRKMLPRGKT--EVEP-D-

---RSVTPDPPKVLKVFNRAILFSAVSRGDARALEGLLEYLLGTDKRLTDEEFKEPSTGK

TCLPKALLNLFGGRNDTIPFLIDVAERTGNLREFVNTPFRDVYYRGQTALHIAIERRCKH

YVELLVEKGADVHAQARGRFFQPKDEGGYFYFGELPLSLAACTNQPDMVHYLTENGHKAA

DLRRQDSRGNTALHALVHIADNTRENTRFLTKMYDLLLVKCARLHPDCSLEDVLDSDGMS

PLMMAAKLGKIGVFQHIIRREIKDEDTRHLSRKFKDWAYGPVYSSLYDLSSVDTCGEEVS

VLEILVYNSRIENRHEMLAVEPINELLRAKWQKFAAATFYISVVSYLATMIIFTLVAYYR

PSGGKLPYTYITSTDYLRLTGEVVTLFSGIFFFITNIKDLFLKKCPGVNSLFIDGSFQLL

YFVYSVLVLVTAALYLSGIEAYLCVMVFALVLGWMNTLYFTRGLKLTGTYSIMIQKILFK

DLFRFLLVYVLFMIGYASALASLLPACPGPGEPCP--PAGC--PTYPECRDTQTFSRFLL

NLFKLTIGMGDLDMVENTRYPVVFLILQVTYIILTFVLLLNMLIALMGETVDQVSKESKK

IWKLQWATTILDIERSFPVCLRKSFRAGEMVTVGKNWDGTPDRRWCFRVDEVNWSHWNQN

LGIINEDPGKN--ETYQ-QG-IQQTARGLRRDRWSTVVPRVVELNKSPP-PGDMVVEM--

-EPLHPGHRE---RPNSLGQH--------------------------------

>Spotted_gar_Trpv4

------------------MTEPEDASG--------PVGSGSGDSGD-SPQENDSSFP-LS

SLANLFEGEEGSPAPE----------------LP--RPGESRQNLRMKFQGAFKKGITNS

MDLLESSIYESPVVPAPKKAPMDSLFDYGTYHQ-PNN-QKRRRKKIPNSKL--AAAS-V-

---KGPAPDPPPVLKVFNRTMLFDAVSRGDTSALQGLLQFLQCNKKRLTDEEFREPSTGK

TCLPKALLNLYSGQNDTIPFLVDIAEQTGNLREFINTPFRDVYYRGQTALHIAIERRCKQ

YVELLVEKGADVHAQARGRFFQPKDEGGYFYFGELPLSLAACTNQPDIVHYLTENSQKKA

DLRRQDSRGNTVLHALVHIADNTRENTRFLTKMYDLLLIKSVKLYPDCNLETVLNNDSMS

PLMMAAKLGKIGVFQHIIRREIKDENARHLSRKFKDWAYGPVYSSLYDLSSLDTCGEEVS

VLEILVYNSRIENRHEMLAVEPINELLRDKWQKFGAVTFYISVVSYLVAMVIFTLVAYYR

PSEGTPPYPYSTSSEYLRMAGEIVTVFTGVFFFLTNIKDLFLKKCPGVNSLFIDGSFQLL

YFIYSVLVLVTAALYLSGIKAYVSVMVFALVLGWMNTLYFTRGLKLTGTYSIMIQKILFK

DLFRFLLVYVLFMIGYASALVCLLHSCPGPDAQCP--EGKC--PTYPQCRDTDTFSTFLL

DLFKLTIGMGDLDMINSTQYPAVFLILLVTYIILTFVLLLNMLIALMGETVGQVSKESKK

IWKLQWATTILDIERSFPVCLRRTFRSGEMVTVGKNSDGTPDRRWCFRVDEVNWSHWNQN

LGIINEDPGKN--ETYQYYG-ISQTVRGLRRDRWSTVVPRAVELHKHPR-SGEAVVEMEQ

FEPLNPGNRP---SRETPNRF--------------------------------

>XM_034925198_Sterlet_Trpv4

------------------MTDPEEPPN--------VAGSGSGDSGD-PQPENDSSFP-LS

SLANLFEGEESAPSPE-----------------P--RPGENRQNLRIRFQDAFKKGISNP

KDLLES--YESPVAPAPKKAPMDSLFDYGTYRQ-PND-NKRRRKKLLRDKR--SVSS-I-

---KGPAPDPPPVLKVFTRLLLFDAVSRGDPSDLQGLLPFLLVHKKRLTDEEFREPSTGK

TCLPKALLNLYGGCNDTIPLLVEISEKTGNLREFINTPFRDLYYRGQTALHIAIERRCKQ

YVELLVEKGADVHAQARGRFFQPKDEGGYFYFGELPLSLAACTNQPDMVHYLVQNAHKKA

DLRRQDSRGNTVLHALVHIADNTRENTRFLTKMYDLLLIKCAKLYPDCNLETVLNNYGMS

PLMMAAKLGKIGVFQHIIRREITDESARHLSRKFKDWAYGPVYSSLYDLSSLDTCGEEVS

VLEILVYNSRVENRHEMLAVEPINELLRDKWRKFGAVTFYISVVSYLVSMVIFTLVAYYR

PSEGTPPYQYKSSTDYLRLAGECITLFTGVFFFLTNIKDLFLKKCPGVSSLFMDGSFQLL

YFIYSVLVLVTAALYLSGINAYVSVMALALVLGWMNTLYFTRGLKLTGTYSIMLQKILFK

DLFRFLLVYLLFMIGYASALVSLLNVCPSSDALCP--GGEC--PTYPLCRDTNTFSTFLL

DLFKLTIGMGDLDMINSAQYPAVFLTLLVTYIILTFVLLLNMLIALMGETVGKVSKESKK

IWKLQWATTILDIERSFPVCLRKTFRSGEMVTVGKGCDGTPDRRWCFRVDEVNWSHWNQN

LGIINEDPGKN--ETYQYDG-IPTSVRGLRRDRWSSVVPRVVEFHKSPR-SEEAVVEMDQ

RDPLLQGNRK---TTDT---WRR-DDTKDLCNCDNQKAAVSHRAGQGESEMRD

>XM_028825072_Reedfish_Trpv4m

------------------MAEPEDPSK----------NLSSAESVD-IHPENDSSFP-LS

SLANLFEGEENVHTPE----------------LP--RHVDNKPNLRMKFQGAFKKGISNP

MDLLEYNIYESPVVPAPKKAPMDSLFDYGTYHQ-PNE-QKRRRKKIPHDKL--KTTT---

---AEPAPNPPPVLKVFNRSLLFDAVSRGNVDDLEGLLSFLQAHKKRLTDEEFREISTGK

TCLPKALMNLYGGHNDTIPLLVDIAEKTGNLREFINTPFRDVYYRGQTALHIAIERRCKQ

YVELLVEKGADVHAQARGRFFQPRDEGGYFYFGELPLSLAAGTNQPDIVHYLTENSHKKA

DLRRQDSRGNTVLHALVHIADNTRENTRFLTKMYDLLLIKSAKMYPDCSLESILNNDAMS

PLMMAAKLGKIGVFQHIIRREIKDENARHLSRKFKDWAYGPVYSSLYDLSSLDTCGEEVS

VLEILVYNSRIENRHEMMAVEPINELLRDKWRKFGAVSFYLSVVSYLVAMIIFTLVAYYH

PSEGTPPYAYQTTLDYLRLTGEIITLFSGIFFFFSNIKDLFQKKCPGVNSLFIDGSFQLL

YFIYSILILVTAALYLSGIKAYVSVMVFALVLGWVNTLYFTRGLKLTGTYSIMIQKILFK

DLFRFLLVYVLFMIGFASALVSLLNVCPSPAPECP--GGQC-----PPCRDNTTFSTFLL

DLFKLTIGMGDMDMISNSQSPAVFLILLISYIILTFVLLLNMLIALMGETVGQVSKESKQ

IWKLQWATTILDIERSFPVCIRKVFRSGEMVTVGKNSDGSPDRRWCFRVDEVNWSHWNQN

LGIINEDPGKS--EPYQYNG-ISHTARGLRRDRWSTVVPRVVELNKNSK-SEETAIPMDQ

FEPLNPNNLR---SSIKPQNWKRLEENKI------------------------

>Ghost_shark_Trpv4

------------------MARPEGKDS-----GRVNNSSIENVEEE-GPPA-SESFP-LS

SLANLFENDESNSTLEVSR------------GALRENGESNKQNLRAKFHGAFKNRMPNP

MDLLESSIYDSSMAPAPKKAPMDSLFDYGTYRQ-SSD-EKRKRKKLPKDK----APK-V-

---KPPAPDPPPVLKVFNRVLLFDIVSRGVVSELDGLLSFLLSQKKRLTDEEFREPSTGK

TCLPKALLNLNNGKNDAIPVLLEIAEKTGNLREFINAPFRDVYYRGQIALHIAIERRCKH

YVELLVEKEVDVHAQARGKFFQPKDEGGYFYFGELPLSLAACTNQPDIVHYLTENPHKVA

DLRRQDSRGNTVLHALVAIADNTRENTKFLTKMYDLLLIKCAKLYPDCNLEVILNNDGLS

PLKMAAKLGKIGIFQHIIRREIKDESARHFSRKFRDWAYGPVSSSLYDLSAIDTCGEEVS

LLEILVYNGKIENRYEMLAVEPINELLRDKWKKFAAVSFYLSVISYLTAMVIFTLIAYYR

PSDGKPPYPYNTTADFMRMGGEIITVVTAIFFFFTNVKDLFLKKCPGVNSMFIDGSFQLL

YFIYSILVLVSAGLYLAGEESYLGVMVFALVLGWINTMYFTRGLKLTGTYSIMIQKILVK

DLFRFLLVYLLFMIGFTSALVSLLVTCPINEFECDSDSPNCTSSTYPICRDNLSFSKFLL

ELFRLTIGMGDLDVINSAKFPIVFVILLVSYIILTFVLLLNMLIALMGETVGQVSKKSKQ

IWKLQWATTILDVERSFPICLRKAFRSGEMVTVGINSDGTPDRRWCFRVDEVNWSHWNQN

VGIINEDPGRN--DIYQFDGGINQTMGRLRRDRWSTVVPRVVELNKPSR-ADEYVVGM--

-EHISMDHKYGYGQSQRNIHWKRAESHI-------------------------
